# Effects of Pubertal Exposure to Butyl Benzyl Phthalate, Perfluorooctanoic Acid, and Zeranol on Mammary Gland Development and Tumorigenesis in Rats

**DOI:** 10.3390/ijms23031398

**Published:** 2022-01-26

**Authors:** Yanrong Su, Julia Santucci-Pereira, Nhi M. Dang, Joice Kanefsky, Vishnuprabha Rahulkannan, Meardey Hillegass, Shalina Joshi, Hafsa Gurdogan, Zhen Chen, Vincent Bessonneau, Ruthann Rudel, Jennifer Ser-Dolansky, Sallie S. Schneider, Jose Russo

**Affiliations:** 1 The Irma H Russo, MD-Breast Cancer Research Laboratory, Fox Chase Cancer Center-Temple Health, 333 Cottman Avenue, Philadelphia, PA 19111, USA; ju.delbuono@gmail.com (J.S.-P.); mynhidang@gmail.com (N.M.D.); joicekanefsky@gmail.com (J.K.); vishnu.rahulkannan@nih.gov (V.R.); meardeyso@yahoo.com (M.H.); shalina.joshi@gmail.com (S.J.); hafsagur@buffalo.edu (H.G.); zhen.chen@pennmedicine.upenn.edu (Z.C.); Jose.russohome098@gmail.com (J.R.); 2 Silent Spring Institute, 320 Nevada Street, Suite 302, Newton, MA 02460, USA; bessonneau@silentspring.org (V.B.); rudel@silentspring.org (R.R.); 3 Pioneer Valley Life Sciences Institute, UMASS Chan Medical School-Baystate, Springfield, MA 01199, USA; jennifer.ser-dolansky@baystatehealth.org (J.S.-D.); sallie.schneider@baystatehealth.org (S.S.S.)

**Keywords:** endocrine-disrupting chemicals, pubertal exposure, mammary gland development, tumorigenesis, RNA sequencing, estrogen signaling, Wnt signaling

## Abstract

Endocrine-disrupting chemicals (EDCs)—including butyl benzyl phthalate (BBP), perfluorooctanoic acid (PFOA), and zeranol (α-ZAL, referred to as ZAL hereafter)—can interfere with the endocrine system and produce adverse effects. It remains unclear whether pubertal exposure to low doses of BBP, PFOA, and ZAL has an impact on breast development and tumorigenesis. We exposed female Sprague Dawley rats to BBP, PFOA, or ZAL through gavage for 21 days, starting on day 21, and analyzed their endocrine organs, serum hormones, mammary glands, and transcriptomic profiles of the mammary glands at days 50 and 100. We also conducted a tumorigenesis study for rats treated with PFOA and ZAL using a 7,12-dimethylbenz[a]anthracene (DMBA) model. Our results demonstrated that pubertal exposure to BBP, PFOA, and ZAL affected endocrine organs and serum hormones, and induced phenotypic and transcriptomic changes. The exposure to PFOA + ZAL induced the most phenotypic and transcriptomic changes in the mammary gland. PFOA + ZAL downregulated the expression of genes related to development at day 50, whereas it upregulated genes associated with tumorigenesis at day 100. PFOA + ZAL exposure also decreased rat mammary tumor latency, reduced the overall survival of rats after DMBA challenge, and affected the histopathology of mammary tumors. Therefore, our study suggests that exposure to low doses of EDCs during the pubertal period could induce changes in the endocrine system and mammary gland development in rats. The inhibition of mammary gland development by PFOA + ZAL might increase the risk of developing mammary tumors through activation of signaling pathways associated with tumorigenesis.

## 1. Introduction

Cancer is the second leading cause of death worldwide according to the Centers for Disease Control and Prevention. In women, breast cancer is the most commonly diagnosed cancer in 159 countries, and is also the leading cause of cancer death in 110 countries [1]. There was a steady increase in the rate of new cases of female breast cancer from 1975 to 2018, according to the cancer trends progress report from the National Cancer Institute [2].

The breast is a hormone-sensitive organ. Changes in circulating or gland-tissue-localized hormones, receptors, or metabolism can have an impact on mammary tissue. Endocrine-disrupting chemicals (EDCs) are a group of chemicals that can mimic or interfere with the body’s hormones. Epidemiological findings support the notion that exposure to environmental pollutants containing EDCs at critical windows during the life course may alter breast development and play a role in changing the susceptibility to breast tumorigenesis [3,4]. The present study aimed to explore whether exposure to the three environmental compounds butyl benzyl phthalate (BBP), perfluorooctanoic acid (PFOA), and zeranol (ZAL) during the pubertal period alters the breast composition and/or the susceptibility to breast cancer. We carried out the animal experiments at Fox Chase Cancer Center (FCCC), and examined the morphology, transcriptome, and tumorigenesis of the rat mammary glands exposed to these compounds. 

BBP is a plasticizer commonly used in construction materials such as vinyl floor tiles, vinyl foams, pipes, coatings, and plastic and rubber products. BBP can be easily released into the environment, where it can enter food via food packaging materials. BBP has been detected in indoor dust, as well as in dietary fats, oils, and infant formulas, at a concentration up to the median of 135 µg/g [5] (Table S1). The European Food Safety Authority (EFSA) panel in 2019 reported that the mean level of estimated daily exposure to BBP through diet for individuals is 0.009–0.207 µg/kg body weight (BW) [6]. The BBP metabolite mBzP was detected in human urine in a US cohort at a concentration of 21.2 ng/mL in samples from 1988–1994 [7], and at 6.8 ng/mL in samples from 2006–2013 [8], suggesting persistent contamination of the diet by BBP. Animal studies have suggested that prenatal and postnatal BBP exposure may affect pubertal onset and mammary gland development in female pups [9,10,11], and may alter sexual differentiation in male rats [12]. Pubertal exposure biphasically altered serum testosterone levels in rats [13]. Exposure in young adult rats impaired metabolism of estradiol [14], resulting in changes in the weight of the uterus, kidneys, liver, and ovaries [15,16].

PFOA is a synthetic organic pollutant; it is extensively used in industrial applications, including food packaging, water-resistant coatings, fire extinguishers, floor wax, nonstick coatings on cookware, and many more. PFOA is extremely persistent in the environment and resistant to degradation; it has been detected in the rivers, house dust, popcorn bags, drinking water, and food, at a wide range of concentrations (Table S2). PFOA has also been detected in human blood samples. Increasing concentrations of PFOA in maternal plasma were associated with low birth weight [17], high inhibin B [18] and triiodothyronine levels in cord blood [19], and overweight female offspring at 20 years of age [20]. High PFOA serum concentrations resulting from occupational exposure significantly increased mortality as a result of mesothelioma [21], liver cancer, liver cirrhosis, diabetes, and other malignancies [22]. Animal studies have suggested that prenatal or pubertal exposure to PFOA at doses from 0.01 to 10 mg/kg/day could induce persistent inhibition of mammary gland development in mice [23,24,25,26,27,28,29,30]. A study conducted in 2010 estimated that daily intake of PFOA via meat and egg consumption was 0.0187 to 0.0197 mg/kg/day in female toddlers at 2–5 years old [31]. 

ZAL is a metabolite of mycotoxin zearalenone, and is also a synthetic nonsteroidal estrogen used as a growth promoter in livestock under the brand name Ralgro. The use of ZAL has been banned in the European Union since 1985; however, it can still be used in the USA, Canada, and Australia. The study of ZAL in humans and animal models is not as extensive as that of BBP and PFOA. ZAL has been detected in meat, water, grains, and other plant products in the USA, Europe, and Asia (Table S3). In adolescent girls, detection of zearalenone, ZAL, and their metabolites was associated with slower growth and pubertal development [32,33]. In adults, the urinary ZAL concentration was positively associated with breast cancer risk [34]. In animal studies, ZAL exposure caused early pubertal onset, stimulated mammary gland development, promoted proliferation of breast epithelial cells, and tended to increase the number of N-methyl-N-nitrosourea (MNU)-induced mammary tumors per rat [35,36,37,38,39]. The estimated maximum daily ZAL exposure was 5.8–7 mg for ZAL production workers (0.08–0.1 mg/kg for a worker with 70 kg BW) in a study conducted in 1989 [40]. The lowest ZAL dose used in the reported animal studies was 0.1 mg/kg/day [36]. 

In addition, EDCs are usually present in the environment as mixtures of low doses. Exposure to mixtures of EDCs may induce additive or synergistic effects that can cause significant adverse effects even when the exposure to a single EDC at such a low dose is not a concern. Therefore, in this study, we also exposed the animals to combinations of BBP, PFOA, and ZAL at low doses, and compared their effects to those of single exposures. 

Here, we present our novel findings on the effects of pubertal exposure to BBP, PFOA, and ZAL—alone or in combination—on the endocrine system, rat mammary gland development, and DMBA-induced tumorigenesis. We analyzed the mammary gland transcriptome and revealed the pathways and important genes that might potentially contribute to the changes in mammary gland development and tumorigenesis induced by the exposure. 

## 2. Results

### 2.1. Effects of Pubertal Exposure to BBP, PFOA, and ZAL on Body Weight, Onset of Puberty, and Estrous Cycling 

Based on our literature review (Tables S1–S3), we chose 0.5 mg/kg/day for BBP_L, 5 mg/kg/day for BBP_H, 0.01 mg/kg/day for PFOA_L and ZAL_L, and 0.1 mg/kg/day for PFOA_H and ZAL_H in our study, in order to mimic the levels of environmental exposure. To examine whether pubertal exposure to the three compounds at the selected doses could be toxic to rats, we monitored signs of toxicity—including hyperactivity, ruffled fur, gait abnormalities, and body weight—during and after the exposure period. There were no significant changes in any of these parameters, except for a slight decrease in body weight at the end of the 21-day treatment in the ZAL_H group (131.8 ± 16.6 g for ZAL_H vs. 144.8 ± 10.1 g for Ctrl, *p* = 0.04) in the D50 study (Figure S1). There was no significant difference in the onset of puberty indicated by the age of vaginal opening (Figure S2). We performed estrous cycling analysis by examining vaginal smears, and no discernable patterns could be drawn from the data. However, when we analyzed the estrous cycle phase of each rat before it was euthanized at 50 or 100 days old, as shown in Figure S3, the BBP_L and BBP_H groups showed relatively lower percentages of rats in the luteal phase (metestrus + diestrus) than the other groups. In addition, the PFOA + ZAL group had the highest percentage of rats in the estrus phase compared to the other groups at 100 days old.

### 2.2. Effects of Exposure on Mammary Gland Whole Mount, Cell Proliferation, and Hormone Receptors 

**Figure 1:**
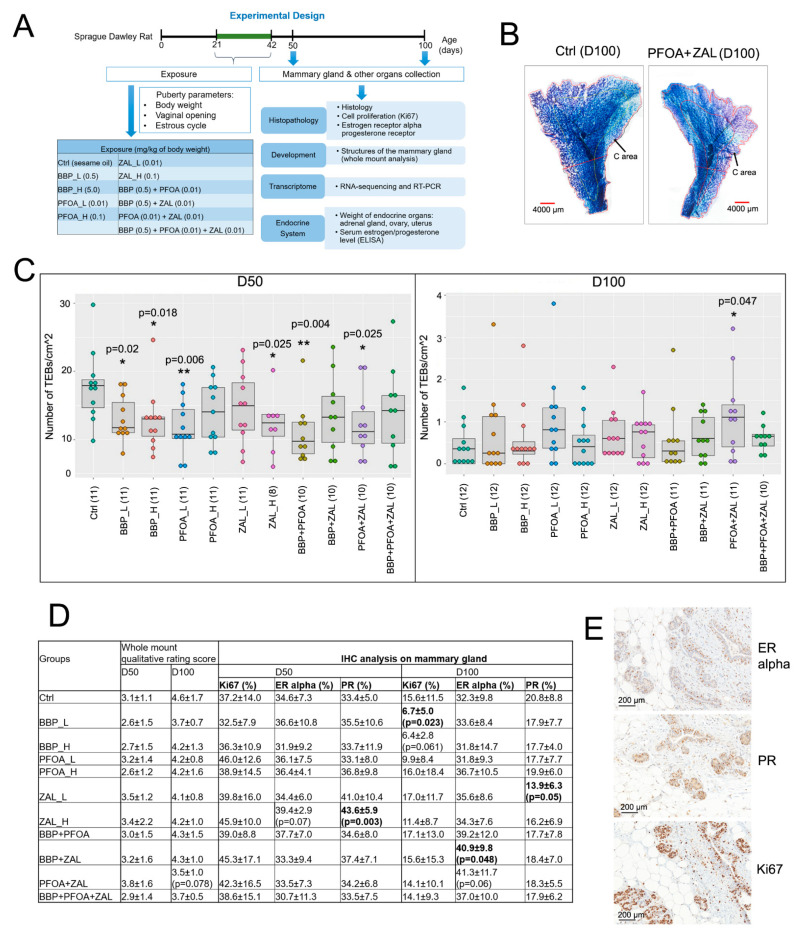
Changes in rat mammary gland development induced by pubertal exposure to BBP, PFOA, and ZAL: (**A**) Experimental design for the study of rat mammary gland development. The rats were treated with 333 µL of working solution per 100 g of BW through oral gavage. (**B**) Representative images of rat mammary glands #4 and 5 whole mounts at an age of 100 days. Scale bar: 2000 µm. (**C**) Boxplots show the number of terminal end buds (TEBs) per cm^2^ in the C area. TEB is a structure with a bulbous tip 80–120 µm in diameter on the perimeter of the gland. Each dot in the graph represents one rat. The samples with poor whole-mount preparation were excluded. Sample size is indicated by the numbers within parentheses after the name of each group; this rule is also applied to other figures. (**D**) Table shows the evaluation of mammary gland development by whole-mount qualitative rating, and IHC analyses of Ki67, ER alpha, and PR in the mammary glands. Data are presented as the mean ± STD. (**E**) Representative IHC images of the rat mammary glands. Magnification: 20× objective. Scale bar: 200 µm. One-way ANOVA or Kruskal–Wallis one-way analysis of variance on ranks were used to compare the phenotypic changes among 11 groups. Two-sample *t*-tests or Mann–Whitney rank sum tests were used to compare treated groups with Ctrl. These tests were also applied to Figure 2; * indicates *p* < 0.05, ** indicates *p* < 0.01 compared to Ctrl.

In order to evaluate the differentiation levels of the mammary glands, we examined the whole mounts prepared from rat abdominal glands #4 and 5 at D50 and D100 (Figure 1A,B); no change in the number of lobules was observed. The number of TEBs in the rats of D50 had a tendency to decrease after exposure to any of the EDCs. Exposure to BBP_L, BBP_H, PFOA_L, ZAL_H, BBP + PFOA, and PFOA + ZAL significantly reduced the number of TEBs compared to controls. However, at D100, most of the exposures did not have an impact on the number of TEBs, except for PFOA + ZAL, which resulted in an increase of the number of TEBs compared to controls, indicating the prolonged presence of the TEBs (Figure 1C). Consistently, qualitative analysis of the mammary gland also showed that the mammary glands of the PFOA + ZAL-treated group had a trend of decreasing differentiation when compared to controls (Figure 1D and Figure S4A).

We next examined the histology of the mammary glands on H&E-stained slides; no significant differences were observed. We then evaluated the mammary epithelial cell proliferation; there was no difference in the percentage of Ki67-positive cells across all groups at D50; however, the percentage of Ki67-positive cells was significantly decreased in the BBP_L, and marginally deceased in the BBP_H group at D100 (Figure 1D). It was reported that the proliferation of mammary epithelium was higher during metestrus compared to the other phases [41]. We then combined all rats independently of EDC treatment and analyzed cell proliferation based on the phase of the estrous cycle. Consistently, mammary glands in the luteal phase (metestrus + diestrus) had a higher cell proliferation compared to the glands in the follicular phase (proestrus and estrus) at both D50 and D100, independently of EDC exposure (Figure S4C).

Both estrogen and progesterone are important hormones regulating mammary gland development via their receptors. We evaluated the expression of ER alpha and PR (Figure 1D,E and Figure S5) in mammary epithelial cells. We observed a trend of increasing percentage of ER-alpha-positive cells in the ZAL_H group at D50 and in the PFOA + ZAL group at D100, as well as a significant increase in the BBP + ZAL group at D100. The expression of PR was similar to that of ER alpha at D50, with only the ZAL_H group showing a significant increase in PR-positive cells, whereas at D100, all treatment groups trended towards a reduction in the number of PR-expressing cells, with a significant decrease in the ZAL_L group in comparison with the controls.

### 2.3. Effects of Exposure on Endocrine Organs and Serum Hormones 

**Figure 2:**
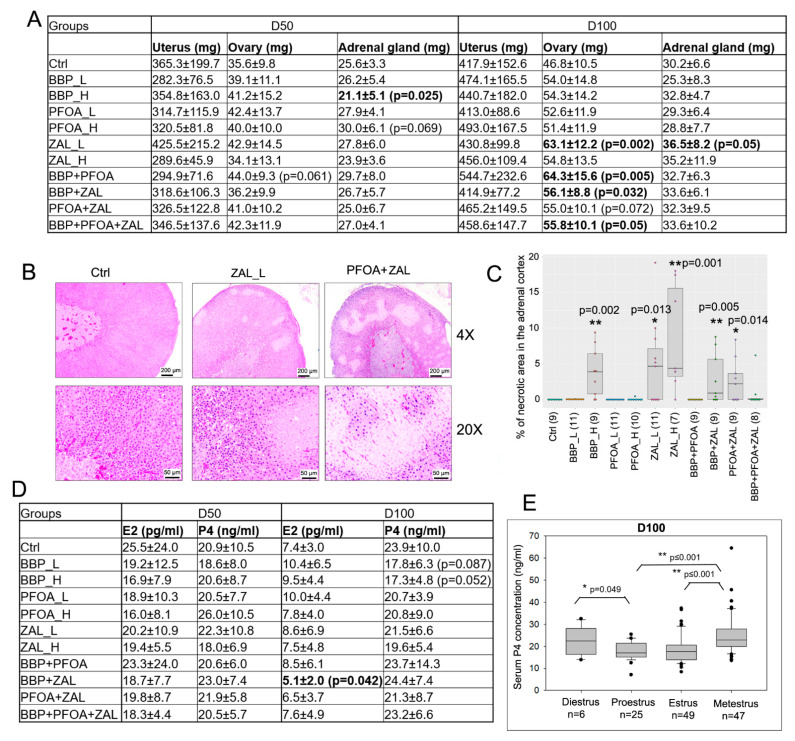
Changes in endocrine organs and serum hormones induced by exposure to BBP, PFOA, and ZAL: (**A**) Table shows the wet weight of uteri, ovaries, and adrenal glands at D50 and D100. The mean weight of two ovaries or adrenal glands of each rat was calculated and used to conduct the analysis. Sample size information is included in the Supplementary Materials. (**B**) Representative images of adrenal glands by H&E staining, showing necrosis in the adrenal cortex. Scale bar: 200 µm for 4×, 50 µm for 20× objective. (**C**) Quantification of the necrotic area in the adrenal gland cortex; each dot represents one rat. The samples with poor sectioning were excluded. (**D**) Serum E2 and P4 concentrations at D50 and D100; data are presented as the mean ± STD. (**E**) Association of serum P4 concentration with the phase of the estrous cycle. Only the samples that had enough cells for vaginal smear analysis were included. The Mann–Whitney rank sum test was used to perform the analysis; * indicates *p* < 0.05, ** indicates *p* < 0.01 compared to Ctrl for (**A**,**C**,**D**).

The endocrine system plays an important role in mammary gland development. We measured the weight of the endocrine organs and examined their histology. Exposure to BBP + PFOA caused a trend of increased ovarian weight at D50 compared to controls. Exposure to ZAL_L, BBP + PFOA, BBP + ZAL, and BBP + PFOA + ZAL caused an increase in ovarian weight, and PFOA + ZAL tended to increase ovaries’ weight, at D100 (Figure 2A and Figure S6A). No significant difference was found by histological examination.

The effects of exposure on adrenal glands were different from those on ovaries. BBP_H significantly decreased the adrenal gland weight, whereas PFOA_H tended to increase it at D50. For the rats at 100 days old, we observed a significant increase in adrenal gland weight by ZAL_L (Figure 2A and Figure S6B). Strikingly, we found necrotic areas in the adrenal gland cortex on H&E slides of 50-day-old rats in the BBP_H group, and in all groups that received treatments containing ZAL. Among these groups, ZAL_H had the highest incidence (6 out of 7 rats) of necrosis. Since there was no necrosis in the BBP_L, PFOA_L, and BBP + PFOA groups, it seems that ZAL was the compound driving the onset of necrosis in the combined treatment groups. In the 100-day-old rats, no necrosis was detected, which could be an indication that the necrosis is a transient effect (Figure 2B,C).

The uterine weight varied across the rats. We did not observe significant changes in uterine weight across different treatment groups (Figure 2A and Figure S7A). The uterine weights from mice in the estrus phase were reported to be higher than those in other phases [42]. Similarly, we observed that the uteri in the estrus phase were heavier than uteri in other phases at both 50 and 100 days old (Figure S7B). These results indicate that the effects of endogenous hormones during the estrous cycle on uterine weight prevailed over the effects of these exposures. 

EDCs such as bisphenol A have been shown to affect serum sex steroid levels [43]. We did not observe any changes in serum estradiol (E2) and progesterone (P4) levels caused by any of the exposures at D50, while at D100, BBP + ZAL exposure significantly reduced E2 levels, and BBP_L and BBP_H had a trend of reducing P4 levels (Figure 2D and Figure S8A,B). Normally, the adult female rats have one serum E2 peak in the proestrus phase and two serum P4 peaks in the diestrus and metestrus phases [44]. We combined rats from all 11 groups and analyzed E2 and P4 levels based on the estrous cycle. Consistently, the E2 level was relatively higher in the proestrus phase compared to the other phases, and the P4 levels in the diestrus and metestrus phases were significantly higher than in the other two phases at D100, although no differences were observed between the four phases of the estrous cycle at D50 (Figure 2E and Figure S9). 

### 2.4. Summary of Phenotypic Changes Induced by Exposure to BBP, PFOA, and ZAL 

Our analyses suggested that although pubertal exposure to low doses of BBP, PFOA, and ZAL did not induce significant changes in body weight or the onset of puberty, it still caused changes in endocrine organs, serum sex hormone levels, and mammary gland development (Table 1). Specifically, the number of mammary gland TEBs was reduced in rats at D50 by many of the treatments. This effect could be due to the TEBs’ regression to terminal ducts or differentiation to other structures [45]. Most importantly, exposure to PFOA + ZAL caused an increase in the TEBs and less developed mammary glands at D100 compared to controls, indicating that PFOA + ZAL had an inhibitory effect on mammary gland development at this age. The prolonged presence of the TEBs might be linked to susceptibility to mammary tumors. In addition, more changes were observed at D100 than at D50, suggesting that the impacts of pubertal exposures can manifest long after exposure. 

### 2.5. Transcriptomic Changes in the Rat Mammary Glands 

**Figure 3:**
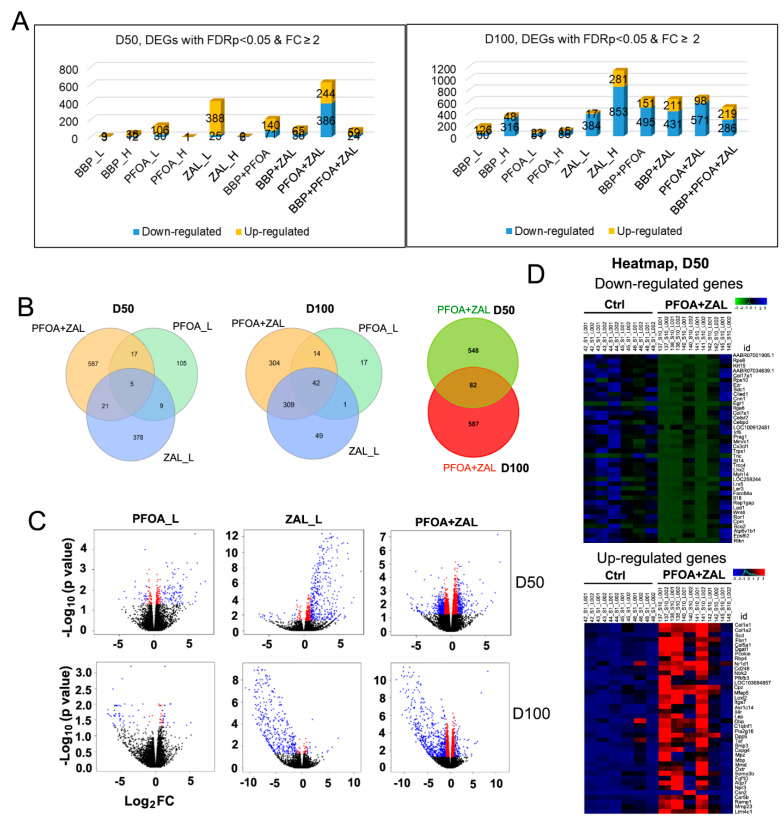
Transcriptomic changes in the rat mammary glands caused by BBP, PFOA, and ZAL exposure: (**A**) The number of DEGs induced by BBP, PFOA, and ZAL -alone or in combination- at D50 and D100; *n* = 6 glands per group. (**B**) Venn diagram representing the number of DEGs in each treatment (FC ≥ 2) and the common genes between/among the treatments. (**C**) Volcano plots of pairwise comparisons, displaying the relation between fold change and significance between the two groups, using a scatterplot view. The *y*-axis shows the negative log_10_ of FDR-adjusted *p*-values (−log_10_(*p* value)); a higher value indicates greater significance. The *x*-axis shows the difference in expression between the treated group and Ctrl, presented in log_2_ fold change (log_2_FC). Each gene is represented by one dot in the graph; red dots represent genes showing statistically significant changes (FDRp < 0.05) and log_2_FC < 1, blue dots represent genes showing FDRp < 0.05 and log_2_FC ≥ 1 (considered as DEGs), and black dots represent non-significant genes. (**D**) Heatmaps of the 40 top down- or upregulated DEGs (FC ≥ 2.0) by PFOA + ZAL compared to Ctrl at D50. Color code for downregulated genes: blue for overexpression, black for intermediate expression, and green for underexpression. Color code for upregulated genes: red for overexpression, black for intermediate expression, and blue for underexpression; *n* = 6 mammary gland samples per group, and each sample was sequenced twice.

We conducted RNA-Seq analysis to elucidate the short- and long-term effects of pubertal exposure to BBP, PFOA, and ZAL on the transcriptomic profiles of the rat mammary glands. After quality checking, filtering, and trimming of the raw reads (Figure S10), the expression value of each transcript was defined, and differentially expressed genes (DEGs) were identified using Robinson and Smyth’s exact test. Figure 3A and Figure S11 show the number of DEGs with FC ≥ 2.0 and FDRp < 0.05. Interestingly, for the changes at D50, PFOA_L and ZAL_L induced greater numbers of DEGs than other individual treatments, and PFOA + ZAL induced the greatest number of DEGs among all treatments. This observation is consistent with the finding that PFOA + ZAL induced more phenotypic changes in the mammary glands at D50 and D100 than other exposures. For the changes at D100, ZAL_H affected many more genes than the other treatments, while PFOA_L, ZAL_L, and PFOA + ZAL still exhibited great numbers of DEGs. In addition, BBP_L, BBP_H, PFOA_H, and all of the combined treatments caused expression changes in more genes at D100 than at D50. In general, more upregulated genes were observed at D50 in the majority of treatments, except for PFOA + ZAL, whereas more downregulated genes were observed at D100 across all treatments, except for the BBP_L group.

Since PFOA + ZAL induced more phenotypic changes in the mammary glands, and also caused significant transcriptomic changes at both D50 and D100, in this report we focused our analyses on PFOA_L, ZAL_L, and PFOA + ZAL (Tables S4–S9). Venn diagrams (Figure 3B) show that at D50, each treatment induced a distinct profile, and there were only five common DEGs among the three groups. At D100, 52.4% of the DEGs of the PFOA + ZAL group overlapped with 87.5% of the DEGs of the ZAL_L group, compared to only 8.4% of the DEGs of the PFOA + ZAL group overlapping with 75.7% of the DEGs of the PFOA_L group, suggesting that ZAL_L exerted a stronger impact on the mammary glands than PFOA_L when the two compounds were combined. In addition, only 13.0% of the DEGs of the PFOA + ZAL group at D50 overlapped with 12.3% of the DEGs of the PFOA + ZAL group at D100.

Volcano plots (Figure 3C) show that PFOA_L induced changes in more genes at D50 (136 DEGs) than at D100 (74 DEGs), whereas ZAL_L (413 DEGs at D50, 401 DEGs at D100) and PFOA + ZAL (630 DEGs at D50, 669 DEGs at D100) had similar impacts on the number of DEGs at the two timepoints. In addition, PFOA_L and ZAL_L induced more upregulated genes at D50, but more downregulated genes at D100. For the PFOA + ZAL group, 61.3% of DEGs at D50 and 85.4% of DEGs at D100 were downregulated genes. In summary, the long-term effects of the three treatments on the mammary gland transcriptomic profiles mainly inhibited gene expression. 

Heatmaps depicting the top 40 (for D50) and 50 (for D100) down- or upregulated genes with the highest or lowest expression for the PFOA + ZAL group are shown in Figure 3D and Figure S12, respectively. As shown in the heatmaps, the two reads of each sample were very consistent. 

### 2.6. Biological Processes and KEGG Pathways Enrichment 

To identify the biological processes (BPs) and pathways enriched in DEGs, lists of up- and downregulated genes were analyzed using the DAVID tool and the Shiny application (Tables S10–S15). At D50, genes upregulated by PFOA_L mainly affected BPs related to muscle systems and KEGG pathways associated with cytokine–cytokine receptor interaction and DNA replication. Meanwhile, at D100, a small number of PFOA_L-upregulated genes were involved in the regulation of developmental processes, cell surface receptor signaling pathways, metabolic pathways in cancer, Rap1 signaling pathways, etc. (Figure S13). ZAL_L appeared to mostly affect processes associated with immune response at D50. Consistently, cytokine–cytokine receptor interaction, chemokine signaling pathways, T-cell receptor signaling pathways, and Th17 cell differentiation were overrepresented in upregulated DEGs. At D100, ZAL_L mainly resulted in downregulation of genes in tissue development, muscle structure development, and muscle system processes, and in pathways including calcium signaling pathways and estrogen signaling pathways (Figure S14). 

**Figure 4:**
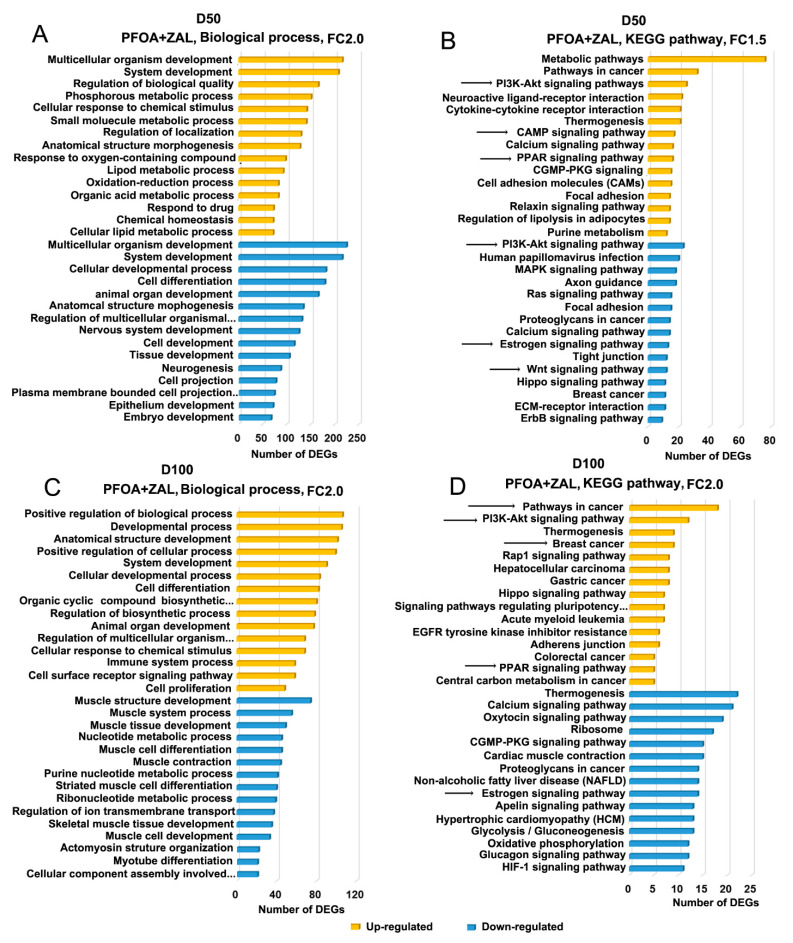
Biological process and KEGG pathway analysis of DEGs induced by PFOA + ZAL: (**A**,**C**) Bar graphs represent genes categorized according to their most prominent biological function. Gene Ontology annotations were extracted using DAVID tools and the Shiny application in R version 3.5.3. (**B**,**D**) Involvement of the KEGG pathway genes was determined using KEGG web tools and the Shiny application in R version 3.5.3. Arrows indicate pathways discussed in the paper.

BPs affected by PFOA + ZAL were very different from those affected by PFOA_L and ZAL_L at D50 (Figure 4A,B). Multicellular organism development and system development were overrepresented in both up- and downregulated genes, whereas metabolic processes were enriched in upregulated genes. Developmental processes such as cellular development, cell differentiation, and epithelium development were enriched in downregulated genes, suggesting that PFOA + ZAL exposure had a marked impact on development. Consistently, KEGG pathway analysis showed that the Wnt signaling pathway, which is important for mammary gland development, was enriched with downregulated genes. Interestingly, for upregulated genes, significant enrichments were found in pathways implicated in tumorigenesis, including PI3K-Akt signaling pathways and PPAR signaling pathways. At D100 (Figure 4C,D), since half of genes affected by PFOA + ZAL overlapped with those affected by ZAL_L, the BPs and KEGG pathways of the downregulated genes were very similar between the ZAL_L and PFOA + ZAL groups, mainly affecting BPs related to muscle systems and pathways such as calcium signaling pathways, CGMP-PKG signaling pathways, and estrogen signaling pathways. For the upregulated genes, PFOA + ZAL affected BPs associated with positive regulation of biological processes, development, and immune system processes. Notably, the KEGG pathways enriched in upregulated genes were mainly associated with tumorigenesis, such as PI3K-Akt signaling pathways, breast cancer signaling pathways regulating pluripotency of stem cells, and PPAR signaling pathways. In addition, PFOA + ZAL induced interaction of PI3K-Akt and PPAR signaling pathways with other signaling pathways in network analysis (Figure S15). 

**Figure 5:**
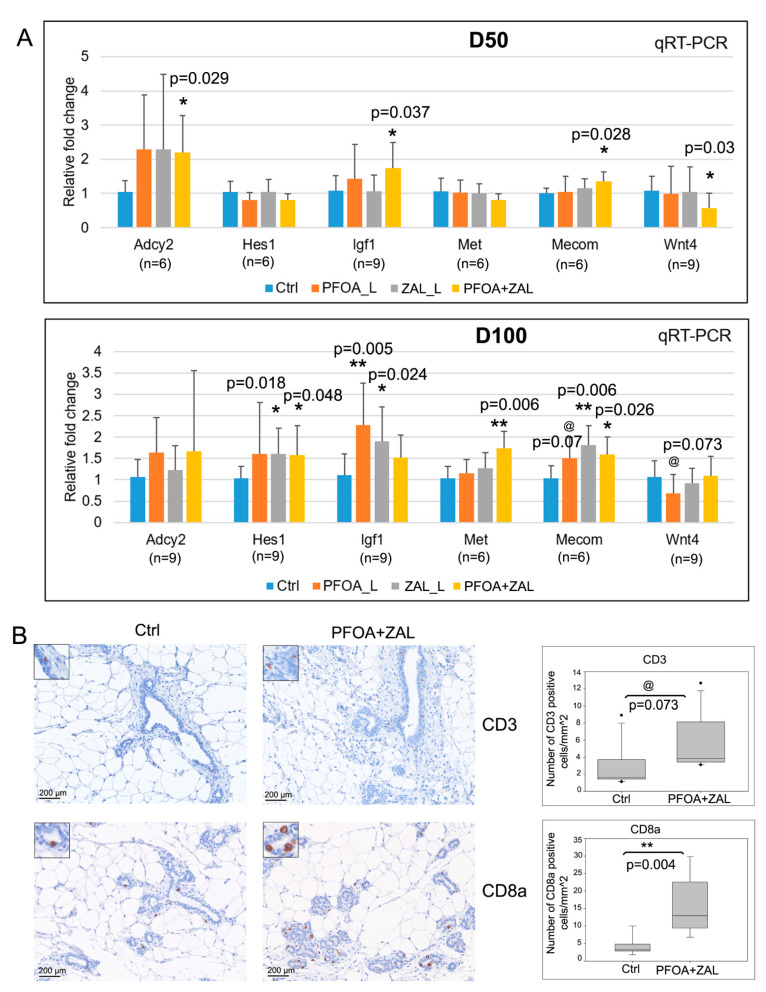
RT-PCR validation and IHC analysis for selected DEGs: (**A**) Graphs show qRT-PCR analysis of selected DEGs involved in mammary gland development or carcinogenesis. (**B**) IHC analysis of CD3 and CD8a in rat mammary glands. The Mann–Whitney rank sum test was used for statistical analysis. Sample size: *n* = 7. Magnification: 20× objective. Scale bar: 200µm; * indicates *p* < 0.05, ** indicates *p* < 0.01, @ indicates *p* < 0.1 compared to Ctrl.

Selected DEGs from the pathways of interest in the PFOA + ZAL group were validated by qRT-PCR (Figure 5A). We confirmed the downregulation of *Wnt4*, the upregulation of *Adcy2*, *Igf1*, and *Mecom* at D50, and the upregulation of *Hes1*, *Met*, and *Mecom1* at D100 by PFOA + ZAL. *Hes1*, *Igf1*, and *Mecom1* were also upregulated by ZAL_L at D100. 

In addition, researchers have shown that exposure to EDCs deregulates adaptive and innate immune mechanisms and interferes with cellular and humoral activities [46]. Therefore, CD3 and CD8a expression in the PFOA + ZAL group at D50—the group that we focused on—were analyzed by IHC as part of a cross Breast Cancer and the Environment Research Program (BCERP) consortia endpoint. The results showed that PFOA + ZAL exposure significantly increased the number of CD8a-positive cells, and tended to increase the number of CD3-positive cells in the mammary gland at D50 (Figure 5B and Figure S16). We further confirmed via qRT-PCR that the RNA expression of *Cd8a* was significantly increased in PFOA + ZAL, and tended to be upregulated in the ZAL_L group at D50 (Figure S17). These results demonstrated an overall impact of exposure to PFOA_L, ZAL_L, and PFOA + ZAL on BPs including development, metabolic processes, muscle systems, the immune system, and on KEGG pathways such as the estrogen signaling pathway, Wnt signaling pathway, and pathways associated with tumorigenesis.

### 2.7. Effects of Pubertal Exposure to PFOA and ZAL on Rat Mammary Tumorigenesis after DMBA Challenge 

**Figure 6:**
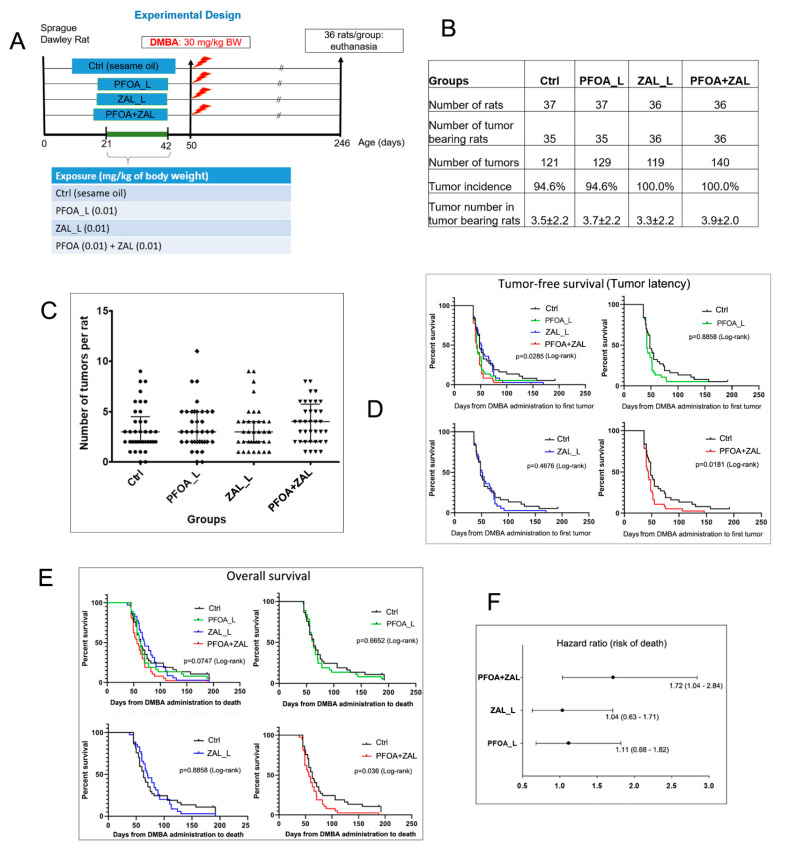
Effects of exposure to PFOA_L, ZAL_L, and PFOA + ZAL on mammary tumorigenesis: (**A**) Experimental design for the mammary tumorigenesis study. Female Sprague Dawley rats were treated with low doses of PFOA, ZAL, or their combination from ages of 21 to 42 days, and were given one dose of DMBA at 30 mg/kg body weight at the age of 50 days. (**B**) Table shows tumor incidence and the numbers of tumors in tumor-bearing rats. (**C**) Dot plots show the number of mammary tumors developed in each rat; each dot represents one rat. One-way ANOVA, *p* = 0.674. The three lines in the dot plots indicate the lower quartile, median, and upper quartile. (**D**) Kaplan-Meier tumor-free survival calculated by the time period from DMBA administration to the appearance of the first tumor, indicating tumor latency. (**E**) Kaplan-Meier overall survival calculated by the time period from DMBA administration to the day of euthanasia. (**F**) Hazard ratio analysis of risk of death using Prism 8.0.

Since PFOA + ZAL exposure inhibited mammary gland differentiation and affected pathways implicated in tumorigenesis, we determined the effects of exposure to PFOA, ZAL, and PFOA + ZAL on the susceptibility of the mammary gland to tumorigenesis. Rats were exposed to the three compounds for 21 days during the pubertal period, and received DMBA at 30 mg/kg BW at 50 days old (Figure 6A). Mammary tumors were observed with a latency of 36 days to 170 days after DMBA administration. Some rats developed multiple tumors. In total, there were 524 mammary masses found from 146 rats, and 510 mammary tumors were confirmed after histopathological analysis. There were no significant differences in tumor incidence or the number of tumors per rat when compared with controls or other treatments (Figure 6B,C). However, rats exposed to PFOA + ZAL exhibited shorter tumor latency (log-rank test *p* = 0.018) and lower overall survival probability (log-rank test, *p* = 0.036) than control rats challenged with DMBA. There were no differences between control and PFOA_L- or ZAL_L-treated rats (Figure 6D,E). Hazard ratio (HR) analysis showed that PFOA + ZAL rats had an HR of 1.72 (1.04–2.84), indicating that this combination increased the risk of death (Figure 6F). Together, these results suggest that pubertal exposure to PFOA + ZAL increases susceptibility to mammary tumorigenesis when challenged with a carcinogen, consistent with the findings of changes in mammary gland development and transcriptomics.

### 2.8. Histopathological and Molecular Subtype Analyses of Mammary Tumors

**Figure 7:**
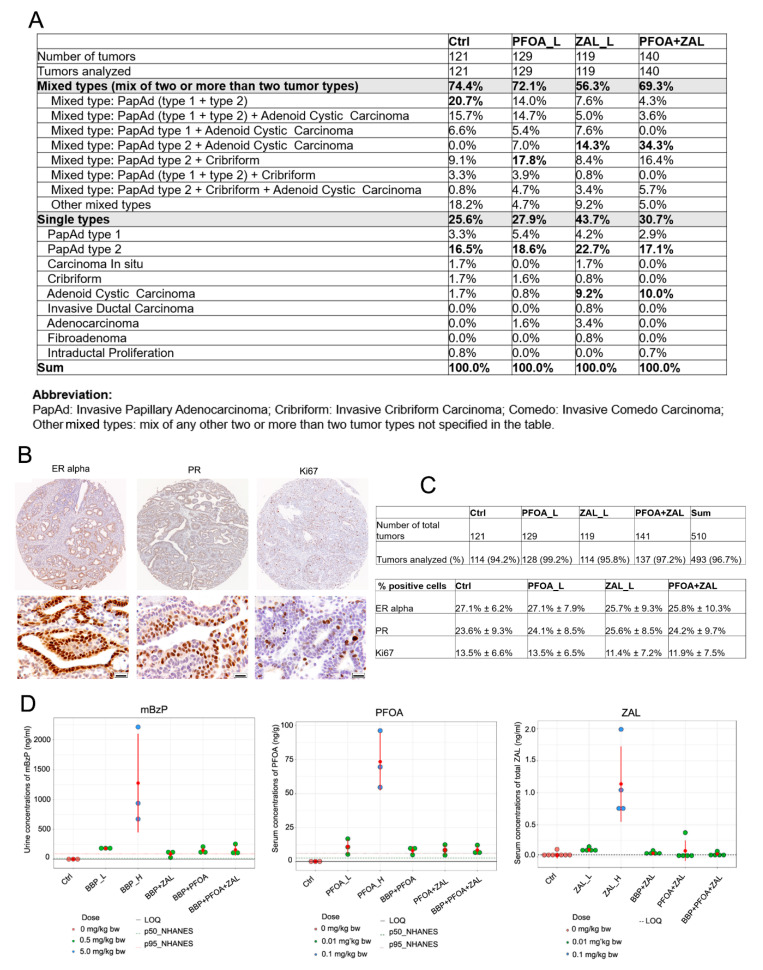
Histopathological analysis and molecular classification of rat mammary tumors: The histopathological type of each tumor was presented as single type when only one histopathological type was found on the tumor, or mixed type when two or more histopathological types were observed on a tumor. (**A**) Histological evaluation of the rat mammary tumors. (**B**) IHC analysis of Ki67, ER alpha, and PR in rat mammary tumors. Upper panel: representative image of one tissue microarray core of each staining. Bottom panel: one high-magnification (40× objective) field showing the staining of the cell nuclei. Scale bar: 20 µm. (**C**) Tables show sample size for IHC analysis and the percentage of positive cells for each staining. Data are presented as the mean ± STD. (**D**) Measurement of the BBP metabolite mBzP in urine, and serum concentrations of PFOA and ZAL at the age of 43 days—1 day after the last administration. NHANES: National Health and Nutrition Examination Survey; LOQ: limit of quantification; p50_NHANES and p95_NHANES represent the 50% and 95% percentile levels in the NHANES 2002–2012 data, respectively.

Histopathological analysis was performed to investigate whether the exposure treatments had any effect on the type of mammary tumors. Interestingly, the control, PFOA_L, and PFOA + ZAL groups developed more tumors of mixed types compared to the ZAL_L group (Figure 7A). The mixture of invasive papillary adenocarcinoma types 1 and 2 was the top mixed type observed for the tumors in control rats. The mixture of invasive papillary adenocarcinoma type 2 and invasive cribriform carcinoma was the top mixed type for the PFOA_L group, while the top mixed type for both the ZAL_L and PFOA + ZAL groups was the mixture of invasive papillary adenocarcinoma type 2 and adenoid cystic carcinoma. For the single-type tumors, invasive papillary adenocarcinoma type 2 was the top type for all four groups. Among all individual types, treatment with ZAL_L and PFOA + ZAL increased the incidence of developing adenoid cystic carcinoma compared to the PFOA_L and control groups. The histopathological similarity between ZAL_L and PFOA + ZAL suggests that ZAL_L had a stronger impact on tumor histopathology than PFOA_L when the two compounds were combined, consistent with the RNA-Seq finding that ZAL_L and PFOA + ZAL had more common DEGs than PFOA_L and PFOA + ZAL.

We previously reported that rat mammary tumors induced by DMBA present similar breast cancer molecular subtypes as those described in human pathology [47]. In order to explore whether PFOA_L, ZAL_L, and PFOA + ZAL played a role in molecular subtype, we evaluated the ER alpha, PR, and Ki67 expression in mammary tumors (Figure 7B,C). Using a cutoff of 1%, except for one tumor from the PFOA + ZAL group (0.79% ER-alpha-positive cells), all tumors were ER-alpha- and PR-positive. There were no significant differences in the proportions of cells positive for ER alpha, PR, and Ki67 among the four groups. 

### 2.9. Measurement of BBP Metabolites and Serum PFOA and ZAL Levels 

In order to enhance the translation of our findings from the rat study, the urine BBP metabolites and serum PFOA and ZAL levels were measured in samples collected one day after the last exposure (D43) (Figure 7D). Urine mBzP concentration (ng/mL) was 180.7 ± 10.5 for BBP_L, 87.2 ± 51.5 for BBP + PFOA, 147.9 ± 53.4 for BBP + ZAL, 155.7 ± 92.2 for BBP + PFOA + ZAL, and 1274.6 ± 825.0 for BBP_H. The average serum PFOA concentration (ng/g) was 11.0 ± 5.7 for PFOA_L, 8.25 ± 3.0 for BBP + PFOA, 8.61 ± 3.8 for PFOA + ZAL, 8.58 ± 3.5 for BBP + PFOA + ZAL, and 73.4 ± 20.9 for PFOA_H. The serum ZAL concentration (ng/mL) was 0.08 ± 0.08 for ZAL_L, 0.056 ± 0.03 for BBP + ZAL, 0.096 ± 0.16 for PFOA + ZAL, 0.046 ± 0.03 for BBP + PFOA + ZAL, and 1.13 ± 0.58 for ZAL_H. 

## 3. Discussion 

Rodents are the most widely used animal models for assessing the risk of exposure to EDCs on mammary gland development and tumorigenesis, because their mammary gland morphology and development process are relatively similar to those of humans. Among rodent models, Sprague Dawley rats are highly sensitive to the DMBA-induced mammary tumorigenesis. In the present study, we exposed Sprague Dawley rats to BBP_L at 0.5 mg/kg/day and BBP_H at 5 mg/kg/day, PFOA_L and ZAL_L at 0.01 mg/kg/day, PFOA_H and ZAL_H at 0.1 mg/kg/day, and the combinations of low doses, for 21 days during the pubertal period. These doses were selected based on the literature published before we started the animal study in 2016, with the intention of mimicking environmental exposure levels. We showed that pubertal exposure to BBP, PFOA, and ZAL induced changes in endocrine organs, serum E2 levels, and mammary gland development in rats. We observed that PFOA + ZAL exposure induced more phenotypic changes in the mammary glands than the other groups, and had the greatest number of DEGs in the mammary gland transcriptome at age D50. We found that BBP_H, ZAL_L, and ZAL_H, as well as all combinations, induced large amounts of DEGs in the mammary glands at D100. We revealed that PFOA + ZAL exposure mainly affected biological processes related to organ development and system development, and signaling pathways associated with mammary gland development and tumorigenesis. We demonstrated that pubertal exposure to PFOA + ZAL increased susceptibility to mammary tumorigenesis when challenged with DMBA. 

The pubertal period is a stage when the mammary gland undergoes dramatic changes under the influence of hormones, growth factors, and other signals. Although we did not observe changes in serum E2 and P4 levels, or in hormone receptors in mammary epithelial cells, as a result of PFOA + ZAL, the number of TEBs in the PFOA + ZAL group was significantly increased at D100 compared to controls. It has been reported that the estrous cycle has an impact on the ductal histo-architecture and lobuloalveolar structure of rat mammary glands [48]; we evaluated whether the number of TEBs was associated with serum E2 and P4 levels, or with the estrous cycle, and there were no associations (Table S16).

For the analysis of serum hormones and uterine weight, in addition to the analysis based on the exposure groups, we also performed analyses in function of the estrous cycle—independently of exposure—due to the limited number of rats in each phase of the estrous cycle per group/timepoint. For the serum hormones, we observed that the physiological peaks of E2 and P4 were disrupted at D50, while they were recovered at D100 (Figure 2E and Figure S9). This suggests that the different compounds might interfere with the production or metabolism of these two hormones shortly after exposure, but the influence fades over time. The analyses of uterine weight (Figure S7B) and Ki67 (Figure S4C) based on the estrous cycle (combining rats from all 11 groups) showed that the effects of endogenous hormones during the estrous cycle on uterine weight and Ki67 in mammary glands followed the expected physiological patterns, suggesting that the EDC doses we used did not disrupt the influence of the estrous cycle on uterine and epithelial cell proliferation in the mammary glands.

To investigate the molecular mechanisms underlying the phenotypic changes in the mammary gland induced by the exposures, we analyzed the mammary gland transcriptome. PFOA + ZAL exposure induced over 600 DEGs above twofold change at both D50 and D100, whereas there were only 82 common DEGs between the two timepoints, suggesting that the treatments induced different changes in the short and long term. Due to the limited number of animals used for RNA-Seq analysis (six per group), we were not able to include serum hormones and estrous cycles into our analysis to assess whether these were influencing the gene expression changes in PFOA + ZAL. However, we evaluated whether these parameters were significantly different among the rats used for RNA-Seq, and we did not observe any differences in E2 or P4 levels, nor in the estrous cycle, between PFOA + ZAL and control rats (Table S17).

The functional analysis of the DEGs from RNA-Seq showed that the estrogen signaling pathway was overrepresented among the genes downregulated by ZAL_L and PFOA + ZAL. Numerous keratin genes—including *Krt15*, *Krt25*, *Krt26*, *Krt27*, *Krt28*, *Krt31*, *Krt32*, *Krt34*, *Krt35*, *Krt36*, and *Krt42*—were downregulated after ZAL_L and PFOA + ZAL exposure. Among these genes, *Krt15*, *Krt25*, *Krt27*, *Krt28*, and *Krt31* served as estrogen-responsive genes, and were downregulated by estrogen in an estrogen-specific mammary gland outgrowth model [49]. In addition, *Krt25*, *Krt27*, *Krt31*, and *Krt32* were downregulated in rat seminiferous tubule culture model by exposure to low-dose bisphenol A [50]—an estrogenic EDC [51,52]. It has been demonstrated that ZAL has estrogenic activity both in vivo and in vitro [53]. Based on these published studies, the finding of downregulation of these estrogen-responsive keratin genes in our study suggests an estrogenic action of ZAL_L and PFOA + ZAL on rat mammary glands. When investigating PFOA + ZAL-upregulated genes at D50, we noted the presence of one important gene—*Adcy2*—in multiple signaling pathways. It was reported that estradiol upregulates *Adcy2* and, subsequently, promotes the production of cAMP in mesenchymal stem cells [54]. *Adcy2* encodes a member of the adenylyl cyclase family, which catalyzes the conversion of ATP to cAMP. The upregulation of *Adcy2* at D50 by PFOA + ZAL (Figure 5A) further suggested the estrogenic action of this combination, and the subsequent influence on the cAMP signaling pathway (Figure 4B) and other pathways.

Among the signaling pathways related to mammary gland development, Wnt signaling is pivotal for morphogenesis of the mammary gland [55]. We observed downregulation of multiple Wnt pathway components—including *Wnt7b*, *Wnt10a*, and *Wnt4*—by PFOA + ZAL at D50. In the pubertal murine mammary gland, *Wnt7b* was exclusively enriched in the TEBs, and was predicted to be a potential regulator of mammary branching development [56]. Wnt10a is one of the mediators of Eda/NF-κB action in developing mammary glands [57]. *Wnt4* is important to progesterone-induced side-branching of the mammary ductal epithelium [58]. We confirmed that *Wnt4* was significantly downregulated by PFOA + ZAL at D50 by qRT-PCR analysis (Figure 5A), suggesting that pubertal exposure to PFOA + ZAL transiently inhibited the Wnt pathway, which is required for mammary gland morphogenesis during the pubertal period and in young adults.

One striking finding of this study is that genes upregulated by PFOA + ZAL at D100 were mainly involved in pathways associated with tumorigenesis. For example, growth factors and growth factor receptors—such as *Igf1*, *Tgfb3*, *Fgf1*, *Pgf*, and *Fgfr3*—and oncogenes—such as *Mecom*, *Met*, *Hes1*, *Myc*, and *Kit*—were found to be upregulated and present in pathways including PI3K-Akt signaling, breast cancer signaling pathways regulating pluripotency of stem cells, PPAR signaling, etc. Igf1 is a growth factor important for postnatal mammary gland development. Mammary gland branching morphogenesis is diminished in mice with a deficiency of Igf1 [59]. *Igf1* is expressed in the mammary gland stroma throughout postnatal development, and in TEBs during pubertal ductal growth, whereas it becomes undetectable in the epithelium after pubertal growth [60,61]. IGF1 is a potent mitogen; the binding of IGF1 to IGF1R and insulin receptors activates the IRS1/Pi3K-AKT/AKB or MAPK pathways, leading to proliferative and anti-apoptotic events [62]. Chronic exposure to IGF1 promoted mammary gland tumor development in the p53R270H/+WAPCre mouse model [63]. Transgenic mice overexpressing Igf1 had increased susceptibility to mammary carcinogenesis when treated with DMBA [64]. We confirmed that the *Igf1* RNA expression was significantly increased at D50 and tended to increase at D100 in the mammary glands exposed to PFOA + ZAL, suggesting that dysregulation of Igf1 expression may contribute to increased susceptibility to mammary tumorigenesis. 

In addition, we validated the overexpression of several oncogenes induced by PFOA + ZAL. *Mecom/EVI1* has been described as a proto-oncogene since its first discovery in 1988 [65]. Mecom is overexpressed in both ER+ and ER- breast cancer. *Mecom* silencing reduces proliferation, apoptosis resistance, and tumorigenicity [66], while overexpression of *Mecom* promotes cell proliferation, migration, and invasion, and induces genes related to epithelial–mesenchymal transition [67]. In this study, *Mecom* was upregulated by PFOA + ZAL at both D50 and D100. *Met* is a proto-oncogene that encodes a member of the receptor tyrosine kinase family. The binding of c-Met with its ligand initiates a series of intracellular signals including PI3K/AKT, Ras/MAPK, JAK/STAT, SRC, and Wnt/β-catenin, and promotes tumor development and progression [68]. Overexpression of MET was associated with basal-like breast cancer [69] in humans, while in a murine model mutationally activated Met induced diverse mammary adenocarcinomas [70]. We demonstrated that *Met* was significantly increased in the mammary glands at D100 by PFOA + ZAL exposure. *Hes1* is one of the Notch signaling target genes. Constitutive activation of the Notch pathway in mammary stem cells and luminal progenitor cells promotes luminal cell commitment and expansion, leading to hyperplasia and tumorigenesis [71]. Notch signaling is aberrantly activated and HES1 is highly accumulated in human breast ductal carcinoma in situ [72]. HES1 overexpression in breast cancer is correlated with advanced stage, node metastasis, negative estrogen receptor expression, and triple-negative status. Upregulation of HES1 promotes cell proliferation and invasion via the AKT pathway and the EMT process [73]. In triple-negative cancer, HES1 functions as an oncogene, and promotes breast cancer stem cells stemness properties via targeting SLUG [74]. In our study, *Hes1* was significantly upregulated by ZAL_L and PFOA + ZAL at D100. Altogether, our study suggests that pubertal exposure to FPOA + ZAL induces upregulation of genes implicated in mammary tumorigenesis.

Although HES1 is associated with triple-negative breast cancer and MET is associated with basal cancer in humans, out of 493 mammary tumors analyzed, we only found one ER-alpha-negative tumor from the PFOA + ZAL group; all others were ER-alpha- and PR-positive. It has been reported by several groups that DMBA-induced mouse and rat mammary tumors are ER^+^/PR^+^ [47,75,76]. The upregulation of *Hes1*, *Met*, and *Mecom* in the mammary gland by exposure to PFOA + ZAL at the doses we used may not be enough to induce mammary tumorigenesis in rats, but it may contribute to the increased mammary tumor susceptibility when challenged with a carcinogen such as DMBA. There could be other drivers for DMBA-induced mammary tumors in the context of PFOA + ZAL; further analysis of the gene mutations and transcriptomics of mammary tumors induced by DMBA after PFOA + ZAL exposure would be necessary in order to identify those drivers.

Furthermore, we noted that pathways related to immune response were overrepresented in ZAL-upregulated genes at D50. For example, the components and the mediators of T-cell receptor signaling—including *CD3d*, *Cd3e*, *CD3g*, *Cd8a*, *CD8b*, *Lck*, and *Zap70*—were upregulated by ZAL. Leukocytes such as macrophages, mast cells, and CD4^+^ T cells have been shown to participate in mammary gland development [77,78]. We demonstrated that the number of CD8a^+^ cells was increased and CD3^+^ cells trended up in the mammary glands of 50-day-old rats exposed to PFOA + ZAL. The upregulation of *Cd8a* in PFOA + ZAL at D50 was further confirmed by RT-PCR. There were no differences in the numbers of CD68+ cells (markers of monocytes and macrophages) and mast cells (Figure S18). The present study is the first report that shows the changes in expression of a great number of genes related to immune response by ZAL, and the increase in the number of CD8a^+^ cells in the mammary gland by PFOA + ZAL. Whether these changes have an impact on mammary gland development and tumorigenesis requires further investigation. We also evaluated whether the number of cells positive for CD3 and CD8a in PFOA + ZAL was related to the estrous cycle or E2 and P4 levels at D50, and no association was found (Table S18).

Finally, we compared the internal BBP, PFOA, and ZAL doses with those reported in NHANES or human studies. The low-dose BBP and PFOA exposure in the present study generated urine mBzP or serum PFOA concentrations close to p95 of the NHANES data. The low-dose ZAL exposure generated serum concentrations in the range of human serum and urine concentrations reported in the available references. This level was 100 times higher than those found in a set of 25 Chilean girls (0.0008 ± 0.0003 ng/mL), whereas it was 5.5 times lower than the concentrations found in 48 adult women (0.437 ng/mL) [32,33,34,40,79,80]. Since PFOA + ZAL exposure had strong effects on mammary gland development and transcriptomics, monitoring PFOA and ZAL exposure in women—especially in occupational workers and women at critical developmental windows—could potentially uncover the association between environmental EDC exposure and breast cancer risk. 

This is the first study to assess the risk of pubertal exposure to BBP, PFOA, and ZAL at environmental levels on rats’ mammary gland development and susceptibility to carcinogen-induced tumorigenesis. We aimed to study the phenotypic and transcriptomic changes of the mammary gland at two timepoints after chemical exposure—50 and 100 days old. Due to the large number of groups studied (11) at the same time, we were not able to increase the number of rats per group, limiting our capability to perform analyses in function of the phases of the estrous cycle for each exposure group. However, the data presented herein are informative, and might contribute to future studies in this field.

In conclusion, we showed that pubertal exposure to low doses of BBP, PFOA, and ZAL that mimic the environmental levels of exposure could exert short- and long-term effects on the mammary gland transcriptome. We found that exposure to the mixture of low-dose PFOA and ZAL inhibited mammary gland development and increased susceptibility to mammary tumorigenesis, which might be mediated by the alteration of estrogen signaling, Wnt signaling, and pathways associated with tumorigenesis. These findings may contribute to the development of molecular targets for intervention, and may also potentially contribute to the implementation of guidelines and regulations for the use of EDCs in industry.

## 4. Materials and Methods 

### 4.1. Chemicals

In this study, BBP (Sigma-Aldrich, #308501, St. Louis, MO, USA), PFOA (Sigma-Aldrich, #171468, St. Louis, MO, USA), ZAL (FUJIFILM Wako Pure Chemical Corporation, #262-01461, Richmond, VA, USA), and DMBA (Sigma-Aldrich, #D3254, St. Louis, MO, USA) were used for treating animals. BBP, PFOA, and ZAL were dissolved in sesame oil (Sigma-Aldrich, #S3547, St. Louis, MO, USA) to make 15 mg/mL, 300 µg/mL, and 300 µg/mL stock solutions, respectively. Working solutions were made freshly before the treatment, and were administered to rats via oral gavage at 333 µL per 100 g BW. DMBA was dissolved in sesame oil at a concentration of 15 mg/mL on the day of administration.

### 4.2. Animals 

Sprague Dawley rats purchased from Taconic Biosciences (Germantown, NY, USA), Inc. were bred in the Laboratory Animal Facility at FCCC. Animal studies were conducted at FCCC using protocol #15–14 approved (28 October 2015) by the Institutional Animal Care and Use Committee (IACUC) of FCCC. Female offspring were weaned until the age of 21 days, and housed in polypropylene cages with water bottles (both of which were polycarbonate/bisphenol A-free) in a temperature-controlled room (23–25 °C) with a 12 h light/dark cycle. An irradiated AIN-93G diet (TD.97184, Harlan Teklad, Madison, WI, USA) was used throughout the experiment to decrease the exposure to phytoestrogens. 

### 4.3. Mammary Gland Development Study

BBP, PFOA, and ZAL were used individually at low and high doses, or combined at low doses. Therefore, there were 10 treatment groups and 1 control group (treated with sesame oil only), with 10 to 12 rats per group. The experiments for the two endpoints (euthanasia at 50 and 100 days old) were designed to study the short- and long-term impacts of the exposure, and were conducted separately due to a large number of animals being studied at a time (Figure 1A). In both experiments, female rats from the same litter were randomized into 11 groups and administered with sesame oil or fresh chemical working solution by oral gavage at the age of 21 to 42 days old (Monday through Friday). Two to three rats from the same treatment group were housed in one cage. All cages of different treatments were maintained in the same room. For the details on monitoring and sample collection, see the Supplementary Materials.

### 4.4. Mammary Tumorigenesis Study

Female Sprague Dawley rats were randomized into 4 groups (36–37 rats/group) and exposed via oral gavage to sesame oil as controls, or to low-dose PFOA (0.01 mg/kg BW) and ZAL (0.01 mg/kg BW)—individually or in combination—at the age of 21 to 42 days old. At the age of 50 days, all rats were challenged with one dose of DMBA at 30 mg/kg body weight via gavage, and the development of mammary tumors was followed for 7 months (Figure 6A). Tumor-bearing rats were euthanized when one of the tumors reached the size of 20 mm in diameter; rats with smaller tumors or without tumors were followed until the criteria were met or until the final timepoint. All mammary gland tumors were collected and fixed in 10% buffered formalin.

### 4.5. Mammary Gland Whole Mount Evaluations

Mammary gland whole mounts were prepared (see the Supplementary Materials). The identifiable structures were on the very perimeter of the gland on whole mount slides. We evaluated the presence of terminal end buds (TEBs), lobule type 1 (Lob 1), and lobule type 2 (Lob 2) in the C-area, which is the space between the perimeter of the gland and 0.4 cm from the perimeter, marked using ImageJ software. Mammary gland differentiation was also evaluated using a qualitative approach (Supplementary Materials). Each whole mount was analyzed blindly by two to three individuals. 

### 4.6. Measurement of Serum Hormones 

Serum was prepared when euthanizing rats at 50 and 100 days old. The Mouse/Rat Estradiol (E2) ELISA kit from ORIGENE (#EA100859, Rockville, MD, USA) and Progesterone (P4) ELISA kit from Enzo Life Sciences (#ADI-900–011, Farmingdale, New York, NY, USA) were used to measure serum E2 and P4 concentrations, following the manufacturer’s instructions. 

### 4.7. Histological Analysis and Mast Cells Staining

Mammary tissues, organs, and tumors were fixed with 10% neutral buffered formalin at least for 24 h, and then processed and embedded in paraffin blocks. Paraffin sections of 4 µm thickness were used for H&E staining and evaluated by Dr. Jose Russo—a senior pathologist. For the analysis of the necrotic area in the adrenal glands, ImageJ was used to trace the necrotic area and the cortex area, and then the percentage necrotic area was calculated. Raw data were collected blindly by at least two individuals. Tissue sections for mast cell analysis were stained with chloroacetate esterase (CAE) (Sigma-Aldrich, #91C-1KT, St. Louis, MO, USA) and analyzed with ImageJ.

### 4.8. Construction of Tumor Tissue Microarray (TMA) 

All H&E-stained mammary tumor slides were reviewed, and areas of interest representing the histopathology of each tumor were circled on the slides by Dr. Russo. The paraffin blocks were retrieved, and the tissues corresponding to the selected areas were used to construct the tissue microarray by using the MTA1-Manual Tissue Arrayer developed by Beecher instruments (Sun Prairie, WI, USA). In total, 24 tissue microarray blocks were constructed, with 1–8 cores per tumor distributed on different blocks. 

### 4.9. Immunohistochemical Analysis (IHC)

Paraffin sections with a thickness of 4 µm were used for IHC; the antibodies used are listed in Table 2. For the details of staining and analysis, see the Supplementary Materials.

### 4.10. RNA-Sequencing (RNA-Seq) 

RNA extraction was performed (see the Supplementary Materials). Six RNA samples with RNA integrity numbers greater than 8.5 from each group were used to perform RNA-Seq. RNA libraries were prepared with TruSeq Stranded mRNA Sample Preparation Kits (#15032611, 15032612, 15032613, 15032619, 20020737, Illumina, San Diego, CA, USA) according to the manufacturer’s protocol, and were sequenced with an Illumina HiSeq2500 sequencing system at the Cancer Genetics Institute of FCCC. In total, we prepared libraries for 132 samples (66 samples for each time point). The samples were barcoded and run in pools using 50 single-ended base pairs. Each pool was sequenced four times to acquire enough coverage for gene expression analyses.

### 4.11. RNA-seq Analysis 

Sequence quality was checked using FastQC (Babraham Institute, Cambridge, UK) prior to alignment. The raw reads were quality filtered and trimmed to reduce positional sequence bias using the Trim sequence tool (CLC Genomic Workbench version 12.0, Piscataway, NJ, USA). Then the cleaned reads were aligned to the Rattus norvegicus (Rnor_6.0) reference genomes (Ensembl rn6) using the CLC Genomics Workbench. The mapping rate ranged from approximately 98 to 99% for all the samples. 

The expression value per gene or transcript was defined by total counts using CLC genomic workbench version 12. A differential expression analysis test (Robinson and Smyth’s exact test) that assumes a negative binomial distribution of the data, and takes into account the over-dispersion caused by biological variability, was used to compare expression levels between each treated group and controls. The genes with absolute fold change (FC) > 1.5 and with a false-discovery-rate-adjusted *p*-value FDRp < 0.05 were considered as differentially expressed genes (DEGs).

In this report, we focused on the analyses of DEGs affected by low doses of PFOA, ZAL, and PFOA + ZAL. We used the DAVID tool and the Shiny application in R version 3.5.3 to identify the biological processes and Kyoto Encyclopedia of Genes and Genome (KEGG) pathways related to the DEGs. Significant terms of Gene Ontology enrichment were determined using Benjamini–Hochberg correction, with cutoff levels of *p* < 0.01. Venn diagrams, volcano plots, and heatmaps were generated using R version 4.0.3 (https://www.r-project.org/, accessed on 20 October 2019), with the packages VennDiagram, ggplot2, and pheatmap.

### 4.12. Quantitative RT-PCR (qRT-PCR) Validation

Genes of interest were validated by qRT-PCR. Probe information is shown in Table 3. For details of the qRT-PCR analysis, see the Supplementary Materials.

### 4.13. Measurement of BBP Metabolites and PFOA, ZAL Serum Levels

We collaborated with Silent Spring Institute for internal dose measurements in the dosed rats. Rat blood serum and urine samples were collected one day after the EDC treatments were completed, at 43 days. Both serum and urine samples were stored in aliquots at −80 °C prior to analysis. The measurement of the BBP metabolite mBzP in urine was conducted at NSF International in Michigan [81]. Serum POFA levels were measured by Dr. Stapleton’s lab at Duke University using a solid-phase extraction combined liquid chromatography–tandem mass spectrometry (LC–MS/MS) method [82]. Total concentrations of ZAL (free + protein bound forms) were measured in serum samples by Dr. Buckley’s lab at Rutgers University using an LC–MS/MS method [33].

### 4.14. Statistical Analysis 

The number of animals was calculated before we conducted the animal study, based on the data from our previous experiments. In brief, for the mammary gland development study, with data from 10 animals per group, we were able to detect an inter-arm standardized effect size of 1.33 with 80% power using a two-sided, two-sample *t*-test. For the mammary tumorigenesis study, with 36 animals in each arm, we had 80% power to detect (a) a difference in tumor incidence of 39% between groups, and (b) an inter-arm standardized effect size of 0.67 for continuous outcomes. One-way ANOVA or Kruskal–Wallis one-way analysis of variance on ranks were used to compare the phenotypic changes among the 11 groups whenever applicable. Two-sample *t*-tests, the Mann–Whitney rank sum test, or Fisher’s exact test were used to compare a specific treatment group with controls. SigmaPlot 12.0 software (Systat Software Inc., San Jose, CA, USA) was used to performed statistical analyses for the mammary gland development study. Associations between TEBs, CD3, or CD8a and endogenous hormones or the estrous cycle were evaluated using linear regression models through the “lm” function of the package “stats” (version 4.1.2) in R (R version 4.1.2). Prism 8.0 (GraphPad Software Inc., San Diego, CA, USA) was used to perform survival analysis, hazard ratios, and comparison of the tumor numbers. SigmaPlot 12.0 was used for the analysis of Ki67, ER alpha, and PR. All statistical tests used a two-sided 5% type I error; *p*-values ≤ 0.05 were considered statistically significant. 

## Figures and Tables

**Table 1:** Summary of phenotypic changes induced by exposure to the EDCs.

Parameters	D50	D100
Body weight	No change	No change
Onset of puberty	No change	No change
TEBs by mammary gland whole mount analysis	Down in BBP_L, BBP_H, PFOA_L, ZAL_H, BBP + PFOA, **PFOA + ZAL**	Up in **PFOA + ZAL**
Mammary gland development by qualitative rating of whole mount	No change	Trend of less developmpent in **PFOA + ZAL**
Mammary gland Ki67 (IHC)	No change	Down in BBP_L
Mammary gland ER alpha (IHC)	Trend of increase in ZAL_H	UP in BBP + ZAL; trend of increase in **PFOA + ZAL**
Mammary gland PR (IHC)	Up in ZAL_H	Down in ZAL_L
Ovarian weight	Trend of increase in BBP+PFOA	Up in ZAL_L, BBP+PFOA, BBP + ZAL, BBP + PFOA + ZAL; trend of increase in **PFOA + ZAL**
Adrenal gland weight	Down in BBP_H; trend of increase in PFOA_H	Up in ZAL_L
Serum E2 and P4 level	No change	Down of E2 in BBP + ZAL
Necrosis in the adrenal gland cortex	Present in BBP_H, ZAL_L, ZAL_H, BBP + ZAL, **PFOA + ZAL**	No

**Table 2:** Antibodies used for IHC.

Antibody	Supplier	Catalogue Number	Dilution
Estrogen receptor alpha (ER alpha)	Santa Cruz	SC-542	1:600
Progesterone receptor (PR)	Santa Cruz	SC-538	1:1200
Ki67	Thermo Scientific	RM-9106-S0	1:200
CD3	Abcam	ab16669	1:100
CD8a	Affymetric eBioscience	14–0808	1:100
CD68	Abcam	ab125212	1:100

**Table 3:** Probes used for qRT-PCR.

Genes	Assay ID	Supplier
*Adcy2*	Rn00578713_m1	Thermo Fisher Scientific
*Hes1*	Rn00577566_m1	Thermo Fisher Scientific
*Hprt1*	Rn01527840_m1	Thermo Fisher Scientific
*Igf1*	Rn00710306_m1	Thermo Fisher Scientific
*Mecom*	Rn01493436_m1	Thermo Fisher Scientific
*Met*	Rn00580462_m1	Thermo Fisher Scientific
*Wnt4*	Rn00584577_m1	Thermo Fisher Scientific
*Cd8a*	Rn00580577_m1	Thermo Fisher Scientific

## Data Availability

All data are provided in the paper. The sequencing data will be provided upon request.

## Supplementary Materials

The following supporting information can be downloaded at: https://www.mdpi.com/article/10.3390/ijms23031398/s1.
