# Supplementary material for: Effects of Pubertal Exposure to Butyl Benzyl Phthalate, Perfluorooctanoic Acid, and Zeranol on Mammary Gland Development and Tumorigenesis in Rats
Source: Int J Mol Sci. 2022 Jan 26;23(3):1398. doi: 10.3390/ijms23031398 (PMC8835802; doi:10.3390/ijms23031398)
Supplement: Supplementary file 1 [file ijms-23-01398-s001.zip › Supplementary figures, 1-17-2022, Final.pdf]

Supplementary Figures

Effects of Pubertal Exposure to Butyl Benzyl Phthalate, Perfluorooctanoic Acid, and Zeranol on Mammary Gland Development and Tumorigenesis in Rats

Yanrong Su[1,4*], Julia Santucci-Pereira[1,4], Nhi M Dang[1,4], Joice Kanefsky[1], Vishnuprabha Rahulkannan[1], Meardey Hillegass[1], Shalina Joshi[1], Hafsa Gurdogan[1], Zhen Chen[1], Vincent Bessonneau[2], Ruthann Rudel[2], Jennifer Ser-Dolansky[3], Sallie Schneider[3], and Jose Russo[1,5*]

Table of Contents

Figure S1. Body weight graphs.

Figure S2. Age of vaginal opening**.**

Figure S3. Estrous phases for rats at 50 and 100 days old.

Figure S4. Qualitative evaluation of rat mammary gland whole mount and cell proliferation.

Figure S5. The impact of exposure on hormone receptors.

Figure S6. Ovarian and adrenal gland weight.

Figure S7. Uterine weight for rats at 50 and 100 days old.

Figure S8. The impact of exposure on serum hormone levels.

Figure S9. Serum E2 and P4 levels analyzed by estrous phases.

Figure S10. Per sequence quality score and per base sequence content.

Figure S11. Number of DEGs at D50 and D100.

Figure S12. Heatmap of the top 50 down- and up-regulated genes by PFOA+ZAL at D100.

Figure S13. Biological processes and KEGG pathways of DEGs induced by PFOA_L.

Figure S14. Biological processes and KEGG pathways of DEGs induced by ZAL_L.

Figure S15. Interaction of PI3K-Akt and PPAR signaling pathways with other signaling pathways induced by PFOA+ZAL.

Figure S16. Positive control for CD3 and CD8a IHC analysis.

Figure S17. RT-PCR for Cd8a.

Figure S18. Analyses of CD68 positive cells and mast cells on rat mammary gland.

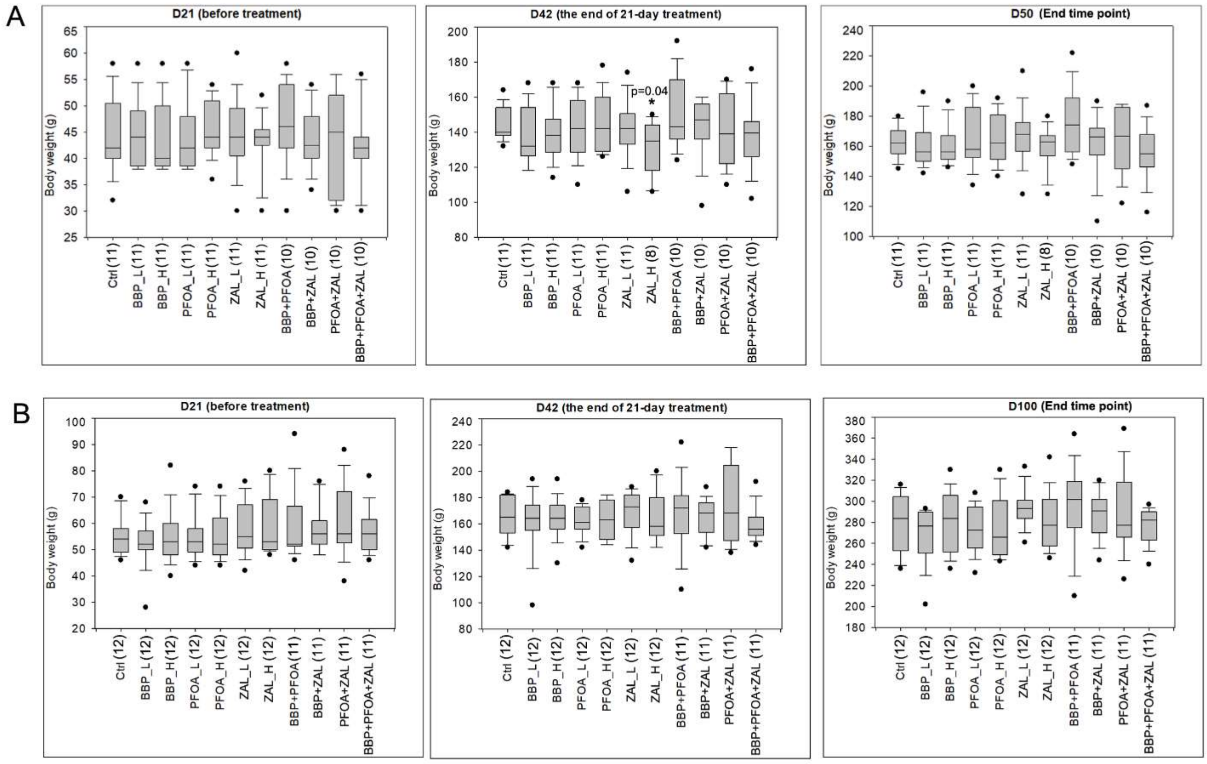

Figure S1. Body weight graphs. Female Sprage Dawley rats were exposed to BBP, PFOA, and ZAL, alone or in combination, during the age of 21 to 42 days. Body weights of the rats were measured three times a week during exposure period and once a week after exposure. Graphs show body weights on the day before treatment, at the end of 21-day treatment, and before euthanasia. (A) Body weight measurements for the D50 study. Three rats in ZAL_H group died at 37 days old due to the malfunction of that cage system. (B) Body weight measurements for the D100 study; Sample size is indicated in the number within parenthese after the name of each group, this rule is also applied to other figures. *indicates $p<0.05$ compared to Ctrl (Two Sample T-test).

A

Age of vaginal openning in the D50 study

Group Name	N	Mean	Std Dev	SEM
Ctrl	11	34.4	2.3	0.7
BBP_L	11	34.1	2.2	0.7
BBP_H	11	34.7	1.8	0.6
PFOA_L	11	34.5	1.4	0.4
PFOA_H	11	34.0	2.0	0.6
ZAL_L	11	34.2	2.1	0.6
ZAL_H	10	34.5	1.9	0.6
BBP+PFOA	10	33.8	2.8	0.9
BBP+ZAL	10	33.3	2.2	0.7
PFOA+ZAL	10	34.1	2.0	0.6
BBP+PFOA+ZAL	10	34.8	1.4	0.4

B

Age of vaginal openning in the D100 study

Group Name	N	Mean	Std Dev	SEM
Ctrl	12	32.7	2.4	0.7
BBP_L	12	34.5	2.8	0.8
BBP_H	12	33.2	1.6	0.5
PFOA_L	12	32.8	1.8	0.5
PFOA_H	12	33.3	2.4	0.7
ZAL_L	12	32.9	2.2	0.6
ZAL_H	12	33.7	2.6	0.7
BBP+PFOA	11	33.0	1.5	0.5
BBP+ZAL	11	33.1	1.5	0.5
PFOA+ZAL	11	33.5	1.8	0.5
BBP+PFOA+ZAL	11	33.9	1.7	0.5

Figure S2. Age of vaginal opening. Vaginal opening of the rats was checked and documented starting from age of 27 days. (A) Age of vaginal opening in the D50 study. (B) Age of vaginal opening in the D100 study.

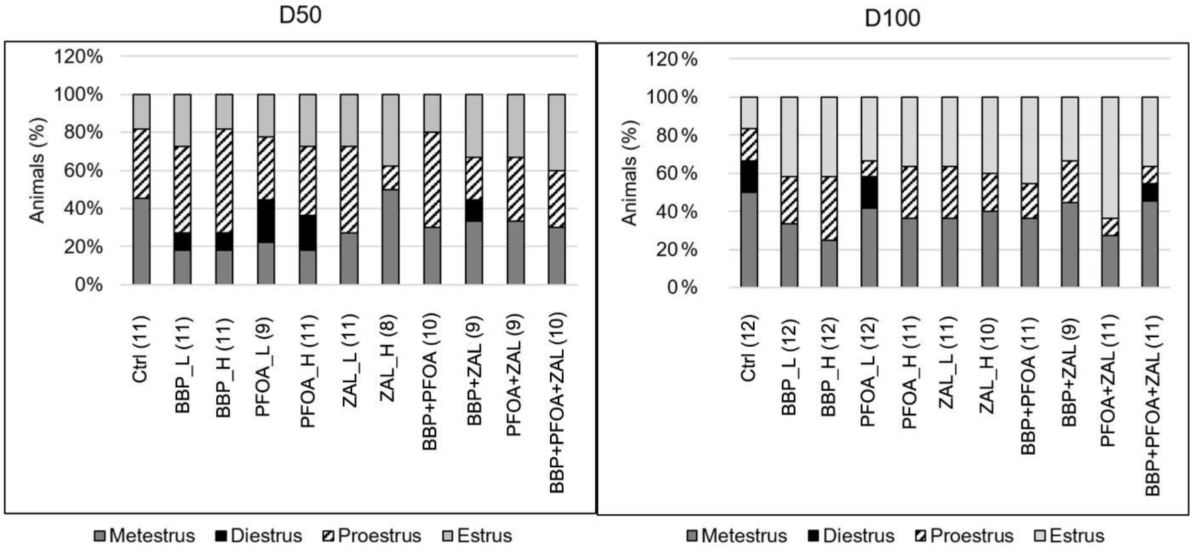

Figure S3. The phases of estrous cycle for rats at 50 and 100 days old. At the two end points for the mammary gland development study, the estrous cycling status of each rat was determined by examinating the cellular composition of the vaginal smear. Samples without enough vaginal smear cells were excluded.

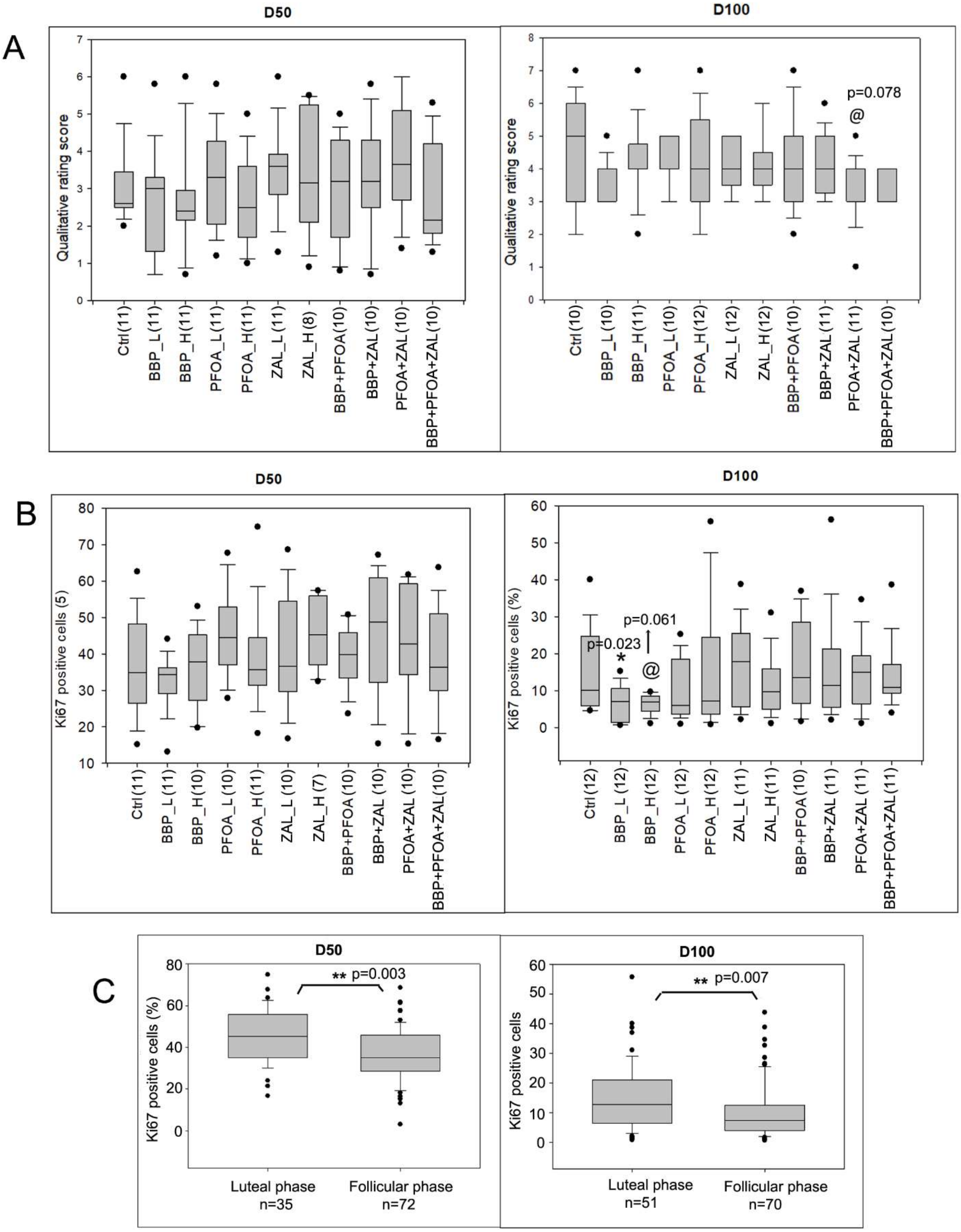
A
D50
Qualitative rating score
Ctrl(11)
BBP_L(11)
BBP_H(11)
PFOA_L(11)
PFOA_H(11)
ZAL_L(11)
ZAL_H(8)
BBP+PFOA(10)
BBP+ZAL(10)
PFOA+ZAL(10)
BBP+PFOA+ZAL(10)
D100
Qualitative rating score
p=0.078
@
Ctrl(10)
BBP_L(10)
BBP_H(11)
PFOA_L(10)
PFOA_H(12)
ZAL_L(12)
ZAL_H(12)
BBP+PFOA(10)
BBP+ZAL(11)
PFOA+ZAL(11)
BBP+PFOA+ZAL(10)
B
D50
Ki67 positive cells (5)
Ctrl(11)
BBP_L(11)
BBP_H(10)
PFOA_L(10)
PFOA_H(11)
ZAL_L(10)
ZAL_H(7)
BBP+PFOA(10)
BBP+ZAL(10)
PFOA+ZAL(10)
BBP+PFOA+ZAL(10)
D100
Ki67 positive cells (%)
p=0.061
p=0.023
*
@
Ctrl(12)
BBP_L(12)
BBP_H(12)
PFOA_L(12)
PFOA_H(12)
ZAL_L(11)
ZAL_H(11)
BBP+PFOA(10)
BBP+ZAL(11)
PFOA+ZAL(11)
BBP+PFOA+ZAL(11)
C
D50
Ki67 positive cells (%)
** p=0.003
Luteal phase
n=35
Follicular phase
n=72
D100
Ki67 positive cells
** p=0.007
Luteal phase
n=51
Follicular phase
n=70

Figure S4. Qualitative evaluation of rat mammary gland whole mount and cell proliferation. (A) Box plots show rat mammay gland development evaluated by qualitative rating of whole mount. Samples with poor whole mount preparation were excluded. (B) Box plots of Ki67 positive cells(%) by IHC analysis on rat mamary gland. Samples without mammary gland structures on the tissue sections were excluded. (C) Association of Ki67 positive mammary epithelial cells with the estrous cycle of the rat when euthanizing. Luteal phase includes diestrus and metestrus phases. Follicular phase includes proestrus and estrus phases. Only the samples that had enough cells for vaginal smear analysis were included. One-way ANOVA or Kruskal-Wallis One Way Analysis of Variance on Ranks were used to compare the phenotypic changes among 11 groups. Two sample T-test or Mann-Whitney Rank Sum Test were used to compare treated group with Ctrl. These tests were also applied to other Supplementary figures. *indicates $p<0.05$, **indicates $p<0.01$, @indicates $p<0.1$ compared to Ctrl.

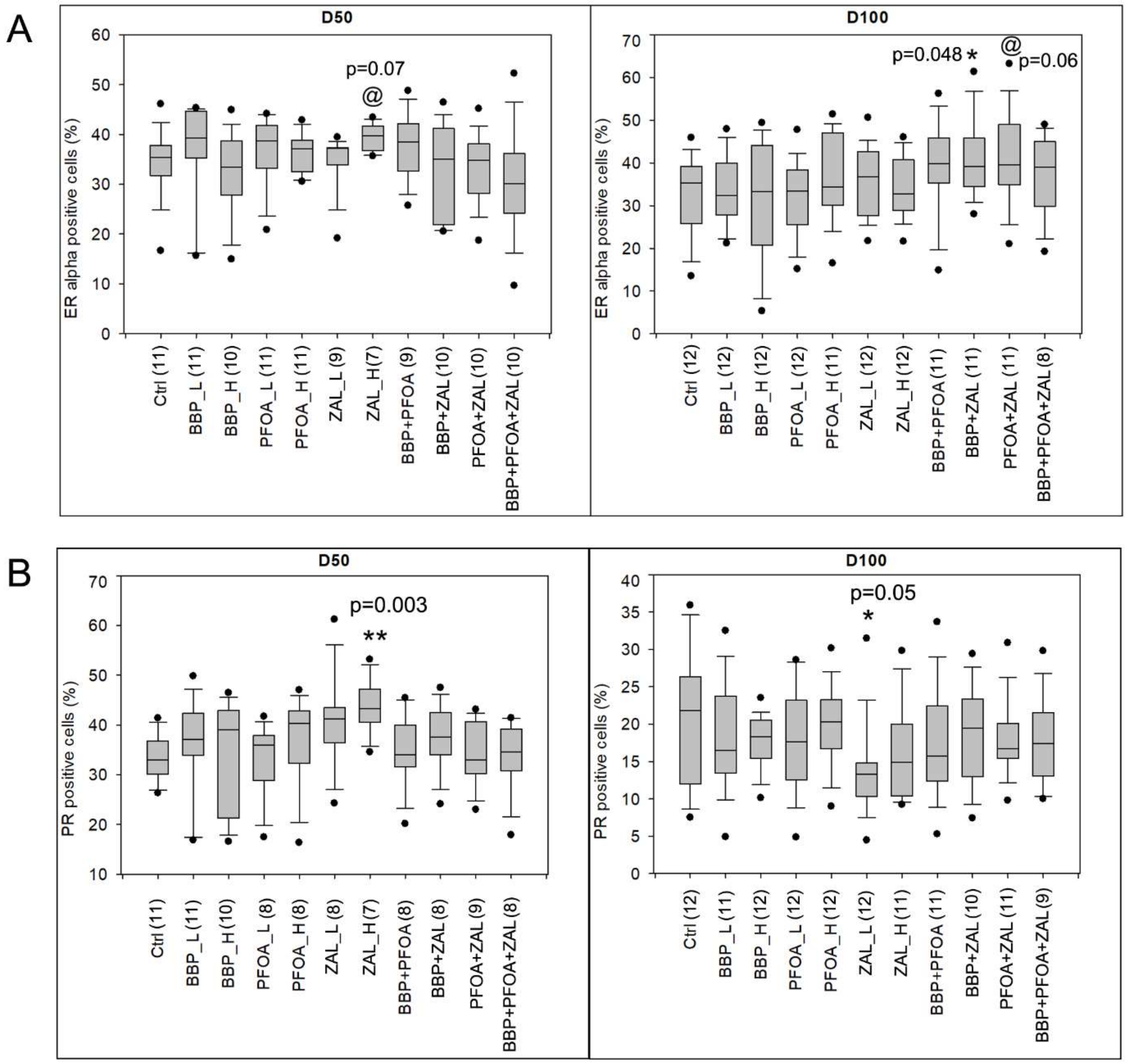

Figure S5. The impact of exposure on hormone receptors. (A) Box plots of IHC analysis for ER alpha expression on mammary gland. (B) Box plots of IHC analysis for PR expression on mammary gland. Samples without mammary gland structures on the tissue sections were excluded. *indicates p≤0.05, **indicates p<0.01, @indicates p<0.1 compared to Ctrl.

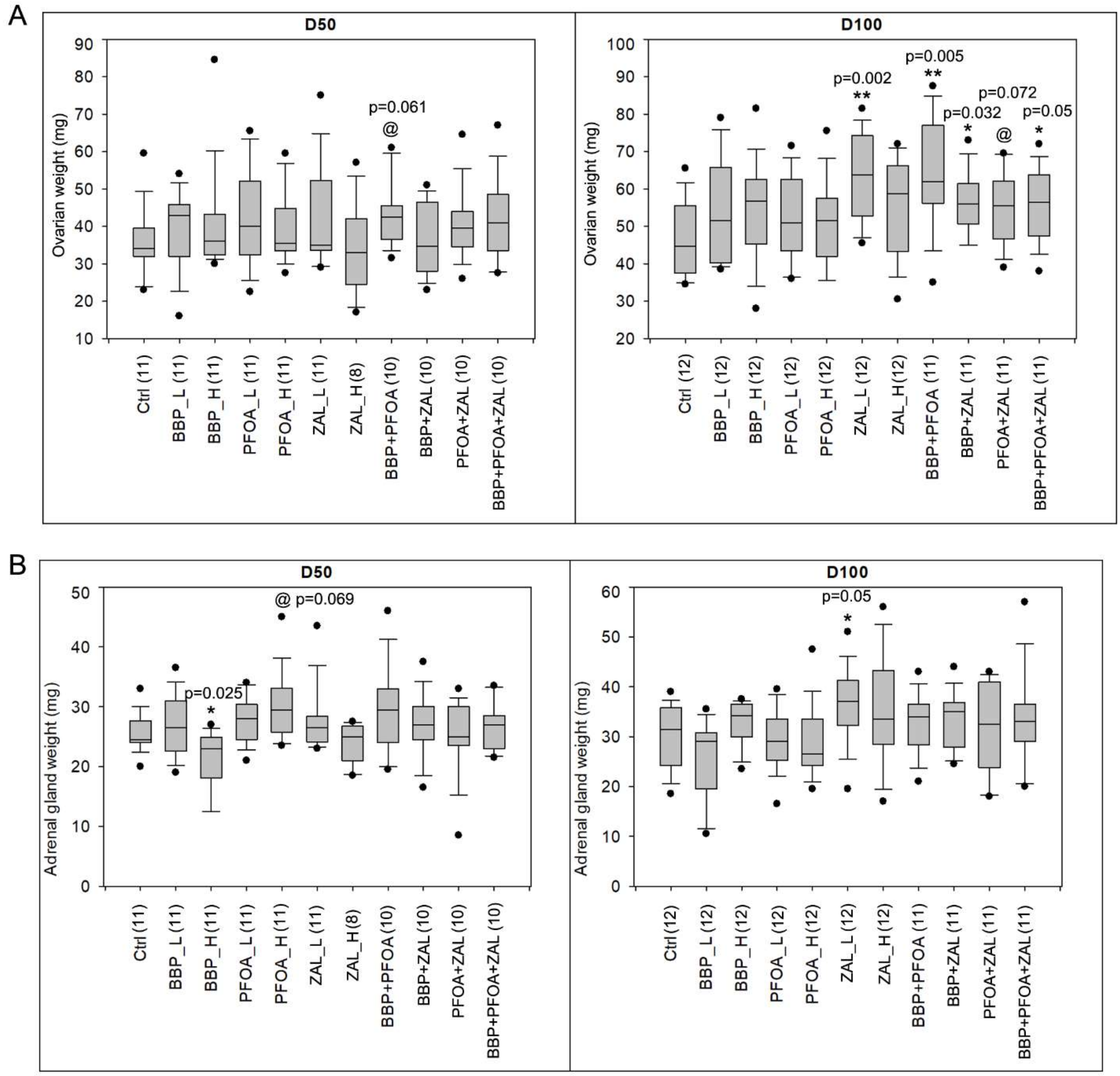

Figure S6. Ovarian and adrenal gland weight. (A) Ovarian weight of rats at 50 and 100 days old. (B) Adrenal gland weight of rats at 50 and 100 days old. Mean weight of two ovaries or adrenal glands of each rat were calculated and used to do the analysis. *indicates p≤0.05, **indicates p<0.01, @indicates p<0.1 compared to Ctrl.

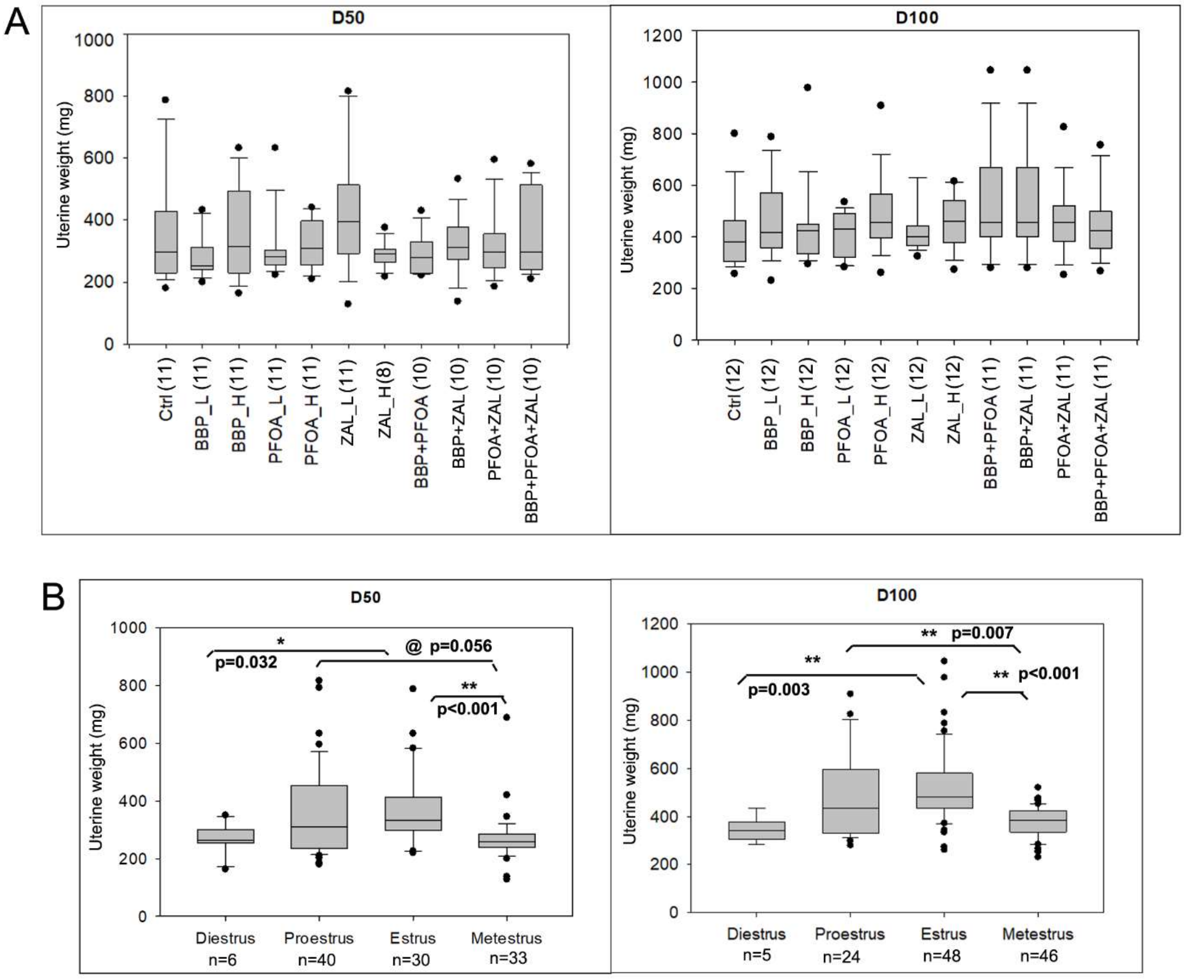

Figure S7. Uterine weight for rat at 50 and 100 days old. (A) Uterine wet weight for each treatment. (B) Uterine weight analyzed by estrous cycle. The box plots show the analysis of uterine weight from all 11 groups independent of treatment. Only the rats with enough vaginal smear cells for evaluating estrous cycle were analyzed. *indcates p<0.05, **indicates p<0.01, @indicates p<0.1 compared to Ctrl.

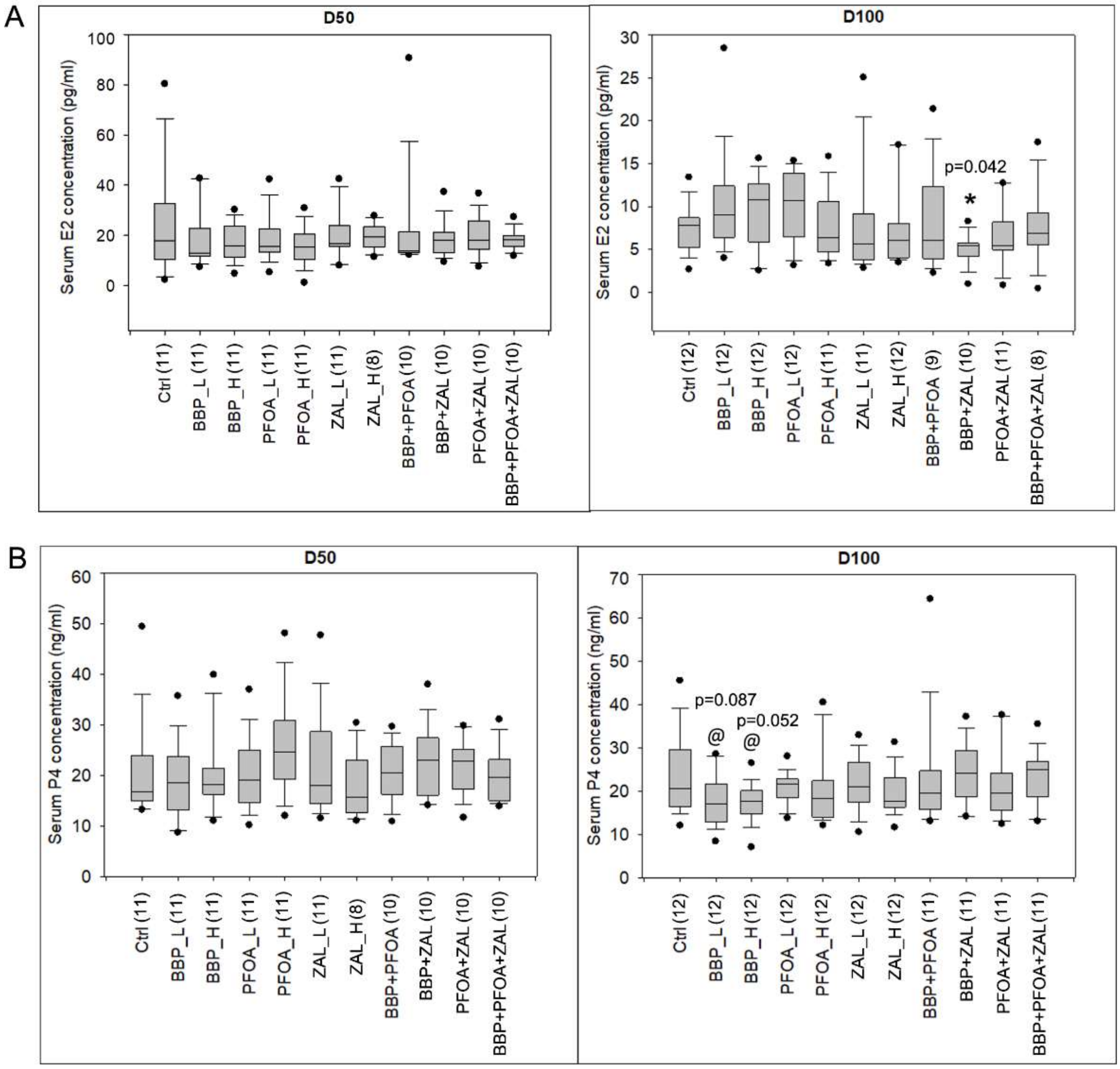

Figure S8. The impact of exposure on serum hormone levels. (A) Serum E2 level measured by ELISA. (B) Serum P4 level measured by ELISA. Only the samples with enough serum were measured. *indicates $p<0.05$, @indicates $p<0.1$ compared to Ctrl.

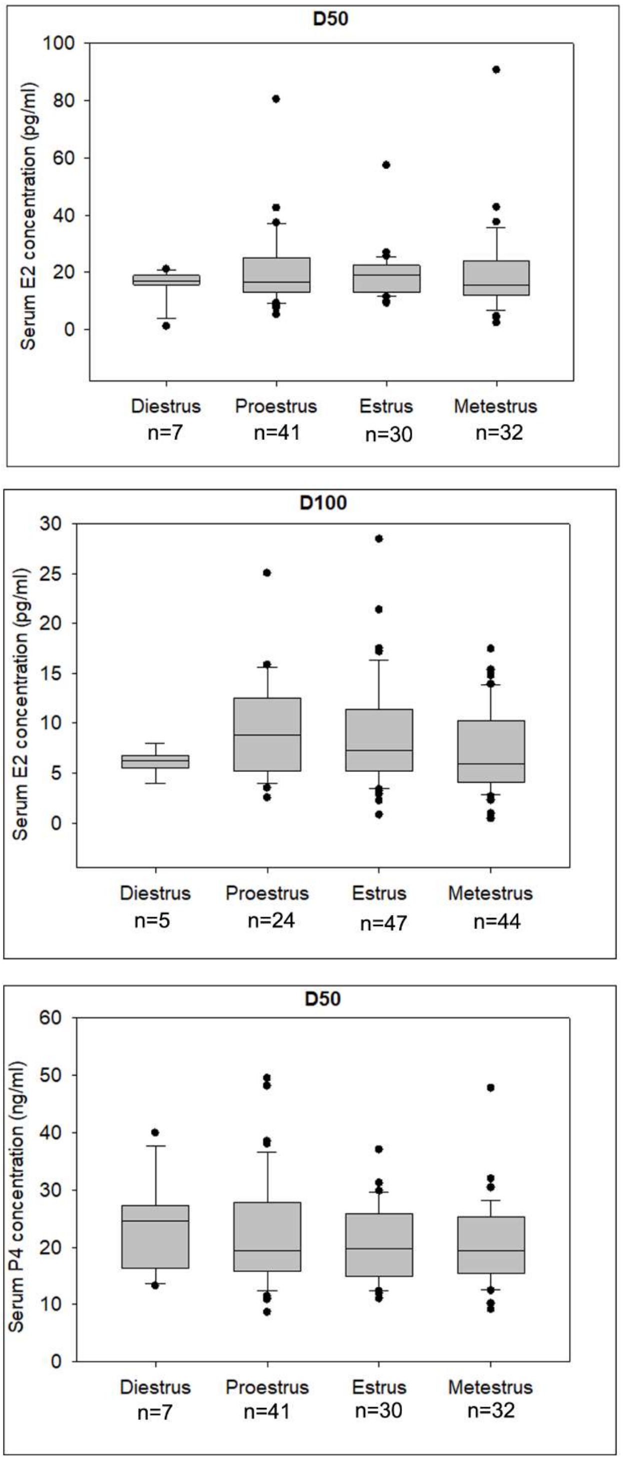

Figure S9. Serum E2 and P4 levels analyzed by estrous cycle. Serum E2 and P4 levels were anlayzed according to the phase of estrous cycle from all 11 groups independent of treatment.

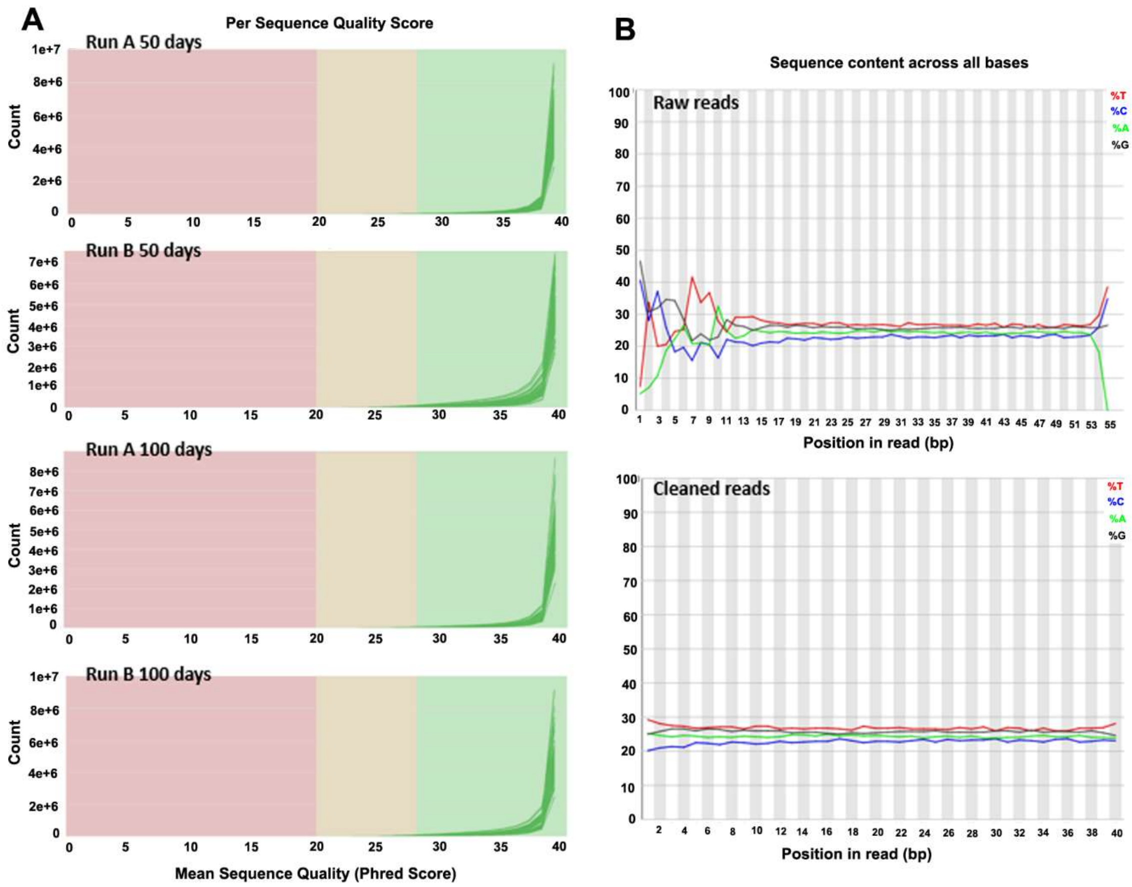

Figure S10. Per sequence quality score and per base sequence content. (A) Overall Phred scores. Each panel on the left-side shows the universal quality values of all reads in each run of two endpoints 50 days and 100 days (528 read files in total). The y-axis shows the total counts corresponding to the Phred scores (x-axis). Green area represents good quality scores. (B) Per base sequence content. Upper panel shows the raw reads with bias and noises for the difference between A and T, or G and C greater than 20% at the first and the end of reads. Lower panel shows the cleaned reads after being filtered and trimmed to reduce the positional bias.

		BBP_L	BBP_H	PFOA_L	PFOA_H	ZAL_L	ZAL_H	BBP+PFOA	BBP+ZAL	PFOA+ZAL	BBP+PFOA+ZAL
D50	Down-regulated	3	12	30	1	25	6	71	36	386	24
D50	Up-regulated	9	36	106	1	388	2	140	65	244	59
	Sum	12	48	136	2	413	8	211	101	630	83

		BBP_L	BBP_H	PFOA_L	PFOA_H	ZAL_L	ZAL_H	BBP+PFOA	BBP+ZAL	PFOA+ZAL	BBP+PFOA+ZAL
D100	Down-regulated	50	316	51	86	384	853	495	431	571	286
D100	Up-regulated	126	48	23	15	17	281	151	211	98	219
	sum	176	364	74	101	401	1134	646	642	669	505

Figure S11. Number of DEGs at D50 and D100. Table shows the number of DEGs (FDRp<0.05, FC≥2) induced by BBP, PFOA, ZAL, alone or in combination, at age of 50 and 100 days, with n=6 glands for each group.

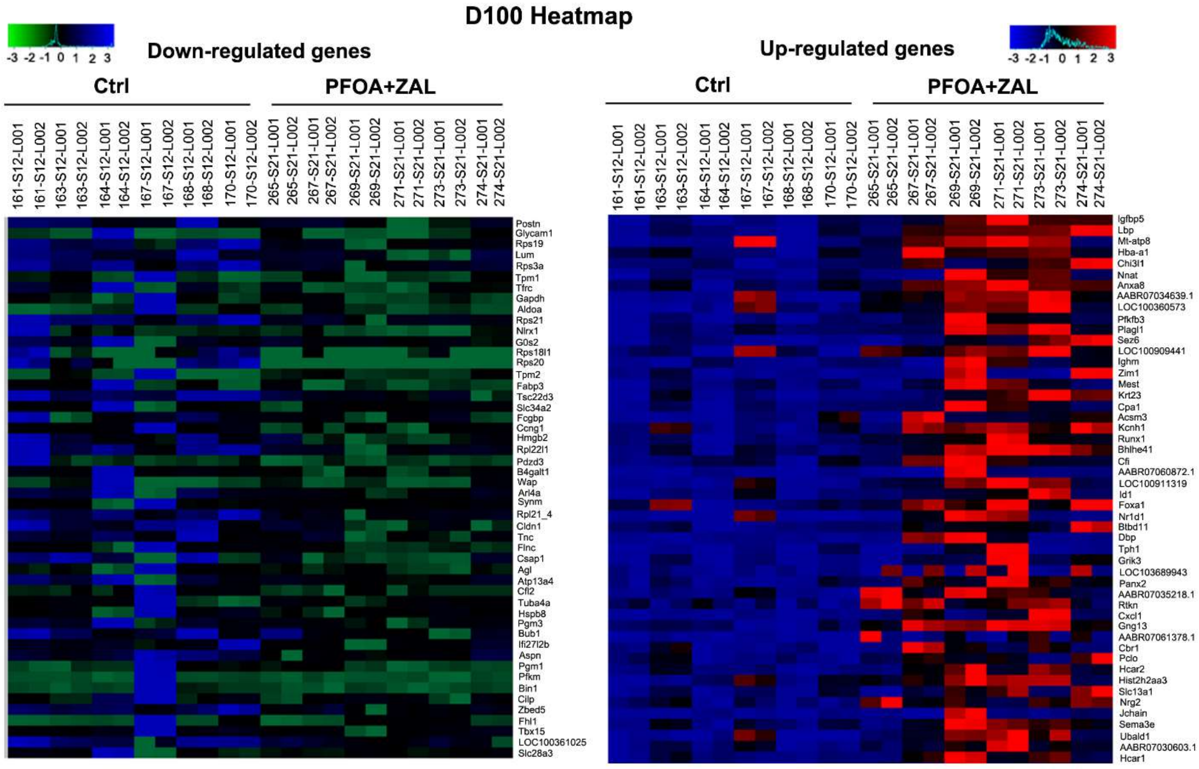

Figures S12. Heatmap of the top 50 down- and up-regulated genes by PFOA+ZAL at D100. Color code for down-regulated genes: blue for over-expression, black for intermediate expression, and green for under-expression. Color code for up-regulated genes: Red for over-expression, black for intermediate expression, and blue for under-expression. N=6 mammary gland samples for each group, and each sample was sequenced twice.

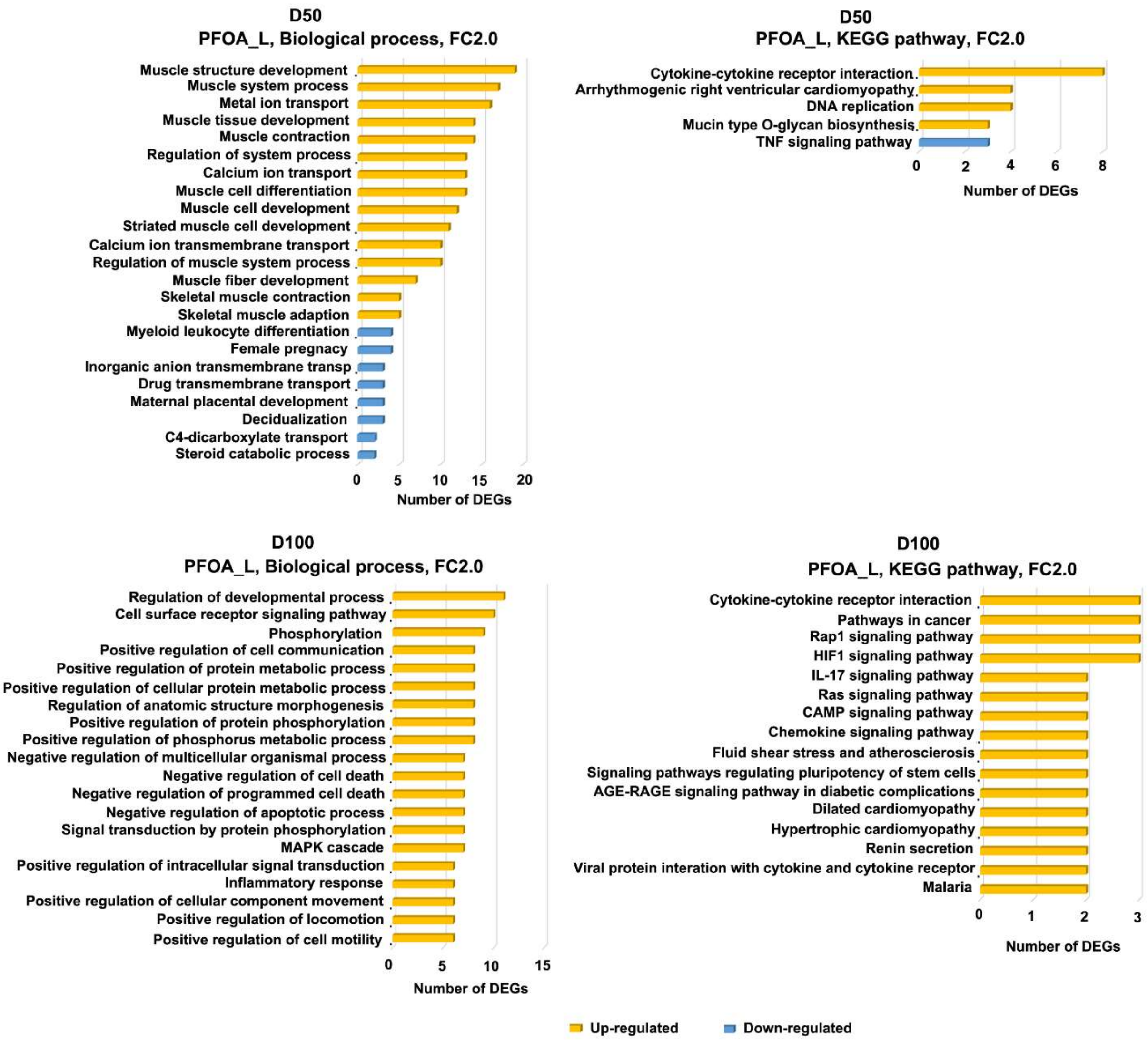

Figure S13. Biological processes and KEGG pathways of DEGs induced by PFOA_L. Bar graph representing genes categorized according to the most prominent biological function. Gene ontology annotations were extracted using DAVID tools and Shiny application in R version 3.5.3. Involvement of the KEGG pathway genes were determined using KEGG web tools and Shiny application in R version 3.5.3.

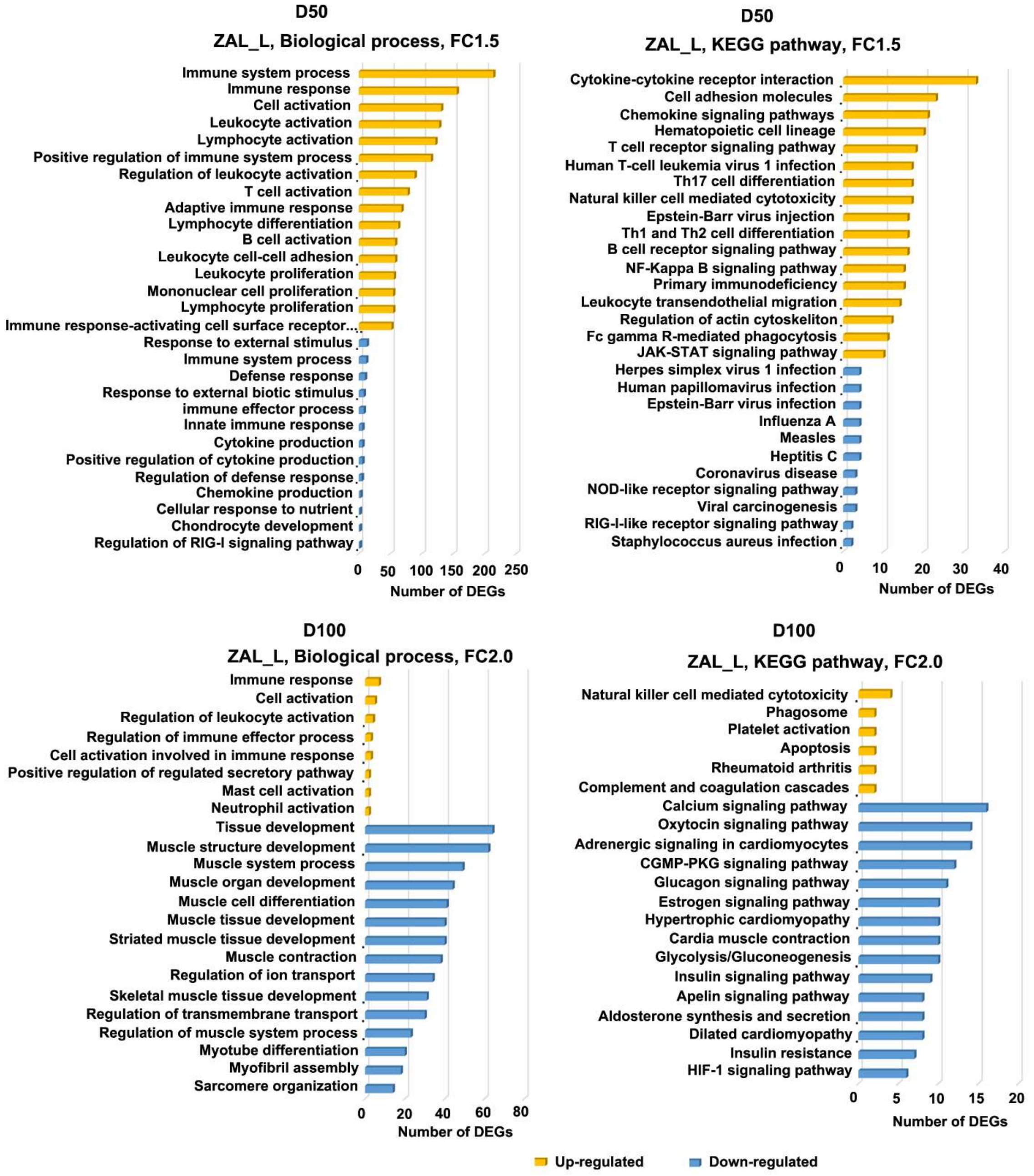

Figure S14. Biological processes and KEGG pathways of DEGs induced by ZAL_L. Bar graph representing genes categorized according to the most prominent biological function.

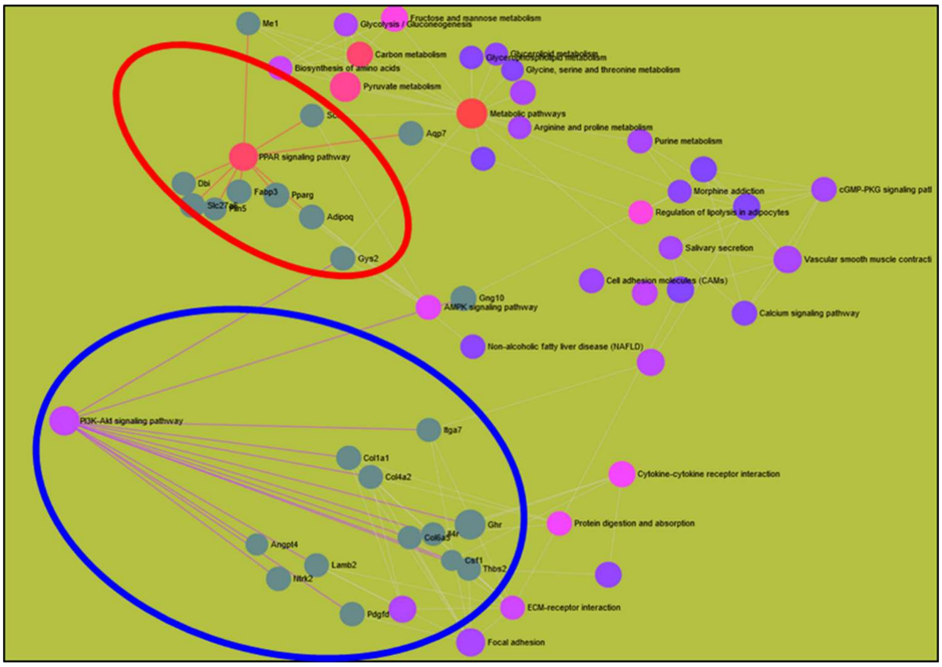

Figure S15. Interaction of PI3K-Akt and PPAR signaling pathways with other signaling pathways induced by PFOA+ZAL. Graph shows the signaling pathways interaction by IPA network analysis.

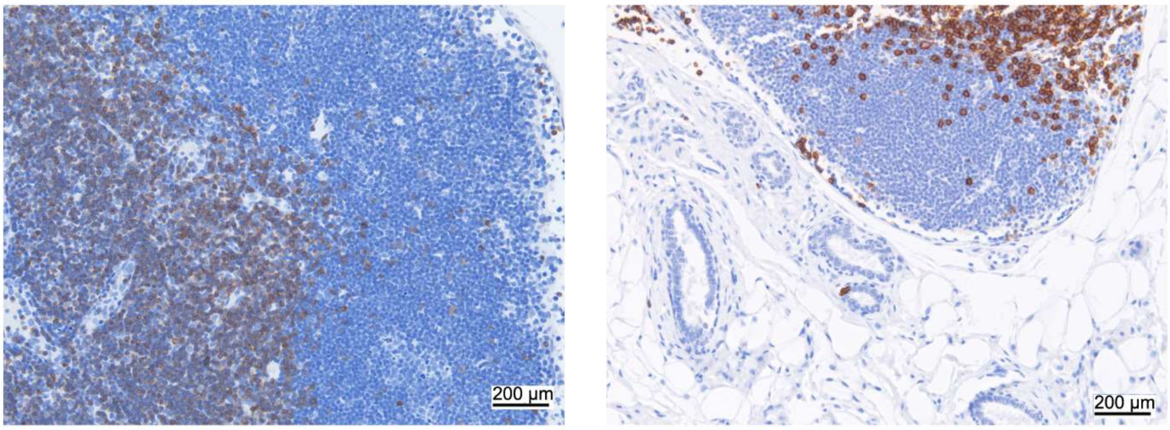

CD3 CD8a

Figure S16. Positive control for CD3 and CD8a IHC analysis. Lymph node in a mammary gland was used as positive control for the staining of CD3 and CD8a. T lymphocytes in the lymph node were positive. Magnification, 20X objective. Scale bar, 200μm.

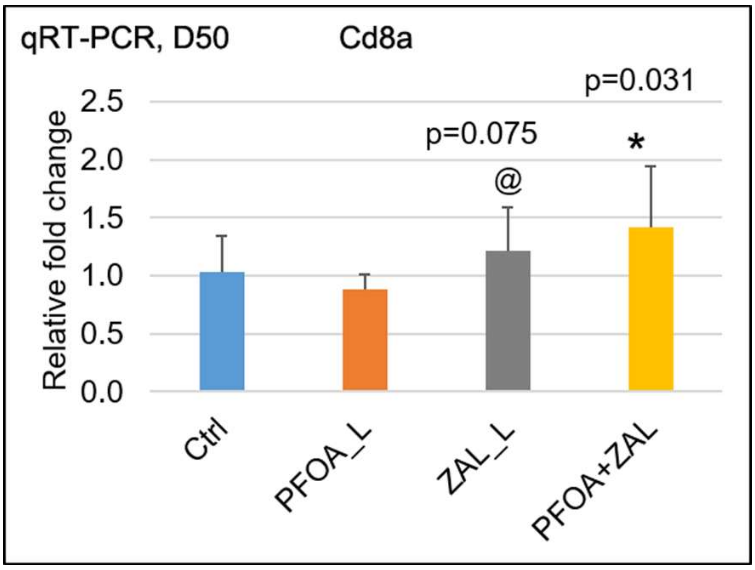

Figure S17. RT-PCR for Cd8a. Graphs show qRT-PCR analysis of Cd8a in the mammary gland of 50 days old rats. Sample size: Ctrl=5, PFOA_L=6, ZAL_L=8, PFOA+ZAL=9. Fisher's exact test was used for the statistical analysis with cutoff 1.2 fold change. *indicates $p<0.05$, @indicates $p<0.10$ compared to Ctrl.

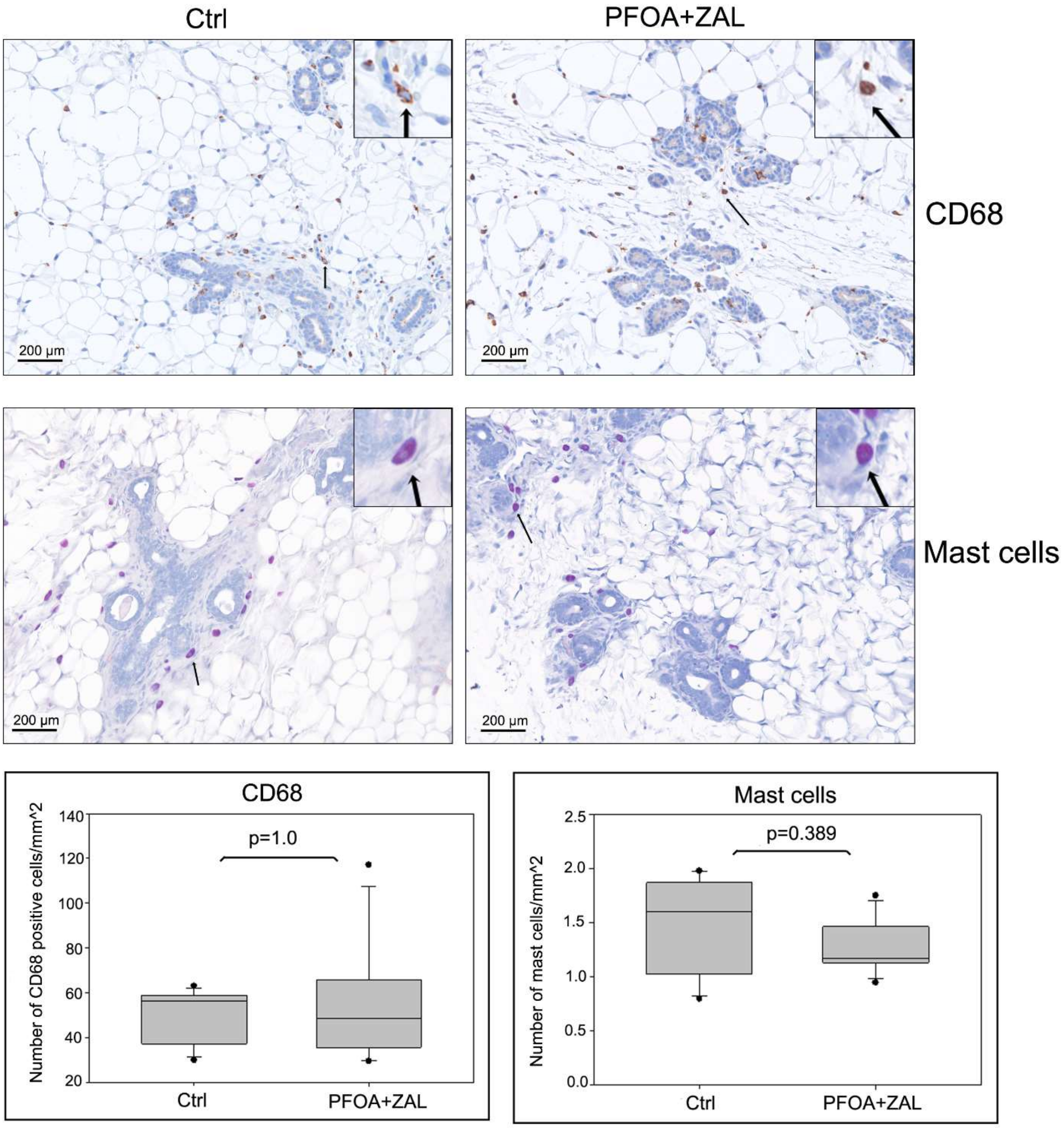

Figure S18. Analyses of CD68 positive cells and mast cells on rat mammary gland. CD68 was analyzed by IHC analysis, mast cells were stained with chloroacetate esterase. Arrow indicates one example of CD68 or mast cells. Sample size: n=7. Magnification, 20X objective. Scale bar, 200µm. Mann-Whitney Rank Sum Test was used for CD68 analysis, and Two Sample t-Test was used for mast cells analysis.
