# Supplementary material for: Effects of Pubertal Exposure to Butyl Benzyl Phthalate, Perfluorooctanoic Acid, and Zeranol on Mammary Gland Development and Tumorigenesis in Rats
Source: Int J Mol Sci. 2022 Jan 26;23(3):1398. doi: 10.3390/ijms23031398 (PMC8835802; doi:10.3390/ijms23031398)
Supplement: Supplementary file 1 [file ijms-23-01398-s001.zip › Supplementary methods, 1-17-2022, Final.pdf]

Supplementary Methods

Vaginal Smears Analysis

The vaginal smears were obtained by pipetting 20 microliters of saline solution into the vaginal orifice of the rat at a depth of approximately 5-8 mm and then flushing for 2 or 3 times. The solution was then taken and deposited onto a slide. After drying, the slide was fixed and then stained with hematoxylin and orange 6 solution (papanicolau staining). The estrous phase was classified into proestrus, estrus, metestrus and diestrus based on the presence and proportion of nucleated cells, keratinized cells or leukocytes.

Monitoring and Samples Collecting for the Mammary Gland Development Study

During the EDC exposure period (21 to 42 days old), body weight of the rats was measured three times a week. Vaginal opening was checked starting from 27 days old, and vaginal smear was performed starting from 40 days old. One day after the last exposure, urine and blood (from tail vein) were collected from these rats (43 days old) to measure the internal concentrations of these compounds or metabolites. During the exposure period, all rats were also monitored for any signs of toxicity. After exposure, the rats were monitored twice a week and euthanized at 50 (referred as D50 in the study) or 100 days old (referred as D100). Mammary glands were collected for whole mount, histological, and RNA analysis. Endocrine organs such as adrenal glands, ovaries, and uterus were collected for histological study. Blood was drawn from the heart to measure the serum hormonal level. The estrous cycling status of each rat at D50 and D100 were also determined via examination of vaginal cytology.

Mammary Gland Whole Mount Preparation and Imaging

Rat mammary gland #4&5 was excised as a pelt and fixed in 10% neutral-buffered formalin for at least 48 hours. Fixed glands were dissected from skin and defatted in acetone for 4 days, rehydrated, and stained with 0.025% toluidine blue (W/V) in McIlvaine buffer, pH 4.0. After staining and dehydration, glands were dissected by removing muscle and stroma, and then mounted with Rihcard-Allan Scientific Cytoseal™ 60 on glass slides. A stereomicroscope and the Zeiss application, ZEN, were used to take pictures of the entire mammary gland at 0.8x magnification. Individual images were stitched together to form an image of the whole gland.

Qualitative Analysis of the Mammary Gland Whole Mount

The qualitative analysis was based on the three parameters: (1) Branch density, a score of 0 was given to glands with the most space between structures/branching and a score of 2 was given to glands with the least amount of space between branches; (2) Differentiating duct ends, a score of 0 was given if there was a low amount of structures present at the end of the branches and 2 was given to a gland with multiple structures; and (3) Branching growth, which defines the numerous offshoots, a 0 was given to the glands with low branching points and 2 was given to the glands with an excessive amount of offshoots.

RNA Extraction

Piece of mammary glands #4&5 was removed and stored in RNAlater® Stabilization Solution (ThermoFisher Scientific, #AM7021) when euthanizing rats from the mammary

gland development study. Samples were stored at 4°C overnight and then in -80°C freezer until use. Lymph node dissection was performed before RNA extraction to avoid contamination of the sample with large amount of immune compounds. Mammary gland tissues (~30 mg) were lysed in QIAzol buffer and homogenized using Precellys 24 tissue homogenizer (Bertin Corp.). The total RNA was extracted by using AllPrep DNA/RNA Mini Kit (Qiagen, #80204) from two different pieces of each sample. RNA quantity was assessed using NanoDrop ND-100 spectrophotometer (Thermo Scientific), and RNA integrity was checked by Bioanalyzer RNA 6000 Nano assay (Agilent, US).

Quantitative RT-PCR (qRT-PCR) Validation

RNA samples were reverse transcribed using High-Capacity cDNA Reverse Transcription Kit (Thermo Fisher Scientific). TaqMan gene expression assays (Thermo Fisher Scientific) were used for the analysis of genes of interest. Twelve to sixteen ng input RNA was used for each reaction in 384 PCR plate with three replicates for each sample. Six to nine samples per group were used for the analysis of each gene. QuantStudio 6 Pro Real-Time PCR system was used to run the PCR, using the program recommend by the manufacturer. Ct threshold values were normalized to the reference gene Hprt1 to calculate the ΔCt value. The $2^{-\Delta\Delta Ct}$ method was used to calculate the relative expression compared to the control group. One-way analysis of variance (ANOVA) and Student's T-test were performed to compare treated and control groups with significance reported for $p<0.05$.

Immunohistochemical Analysis (IHC)

The Super Sensitive™ Polymer-HRP Detection System (BioGenex, #QD430-XAKE) was used to detect the staining of Ki67, ER alpha, and PR and the BioGenex i6000 Autostainer was used to run IHC. The staining of CD3, CD8a, and CD68 was done in collaboration with Dr. Sallie Schneider's group Biospecimen Resource and Molecular Analysis Facility using the DakoCytomation autostainer and the Envision HRP Detection system (Dako, Carpinteria, CA). Each mammary tissue block was sectioned at 4 μm on a graded slide, deparaffinized in xylene, rehydrated in graded ethanols, and rinsed in Tris-phosphate-buffered saline (TBS). Heat induced antigen retrieval was performed in a microwave for 10 (CD68) and 20 minutes (CD3, CD8a) at 98 °C in 0.01 M citrate buffer. After cooling for 20 min, sections were rinsed in TBS and subjected to the primary rabbit monoclonal anti-CD3 [SP7] (1:100, Abcam, ab16669), mouse monoclonal anti-CD8a [XO-8] (1:100, Abcam, ab33786), and rabbit polyclonal anti-CD68 (1:100, Abcam, ab125212) for 30 minutes. Immunoreactivity was visualized by incubation with chromogen diaminobenzidine (DAB) for 10 min. Tissue sections were counterstained with hematoxylin, dehydrated through graded ethanols and xylene, and cover-slipped. Lymph node in a mammary gland was used as positive control for the staining of CD3 and CD8a. Digitalized images were acquired using Vectra® 3 Automated Quantitative Pathology Imaging System (PerkinElmer). For the analysis of Ki67, ER alpha, and PR expression on D50 and D100 mammary glands, collages of mammary epithelial structures on the whole section were created using Paint software. For the analysis of these three markers in mammary tumors, all cells in the TMA cores were analyzed. The number of cells positive for Ki67, ER alpha and PR, and the total cells were analyzed using Aperio ImageScope V11.2.0.780 software (Leica Biosystems Inc., US). Percentage of Ki67, ER

alpha, or PR positive cells was calculated as: (total positive cells/total cells) ×100%. CD3, CD8a, CD68 were counted manually with ImageJ, and data was presented as the number of total positive cells per square centimeter. Raw data was collected blindly by at least two individuals before the statistical analysis using SigmaPlot version 12 (Systat Software Inc., San Jose, CA).
